# Multi‐Omics Revealed the Effects of Intrauterine Hyperglycemia Exposure on the Development of Skeletal Muscle in Offspring

**DOI:** 10.1002/jcsm.70177

**Published:** 2026-01-22

**Authors:** Rui Liu, Junsen She, Xinyuan Li, Yishang Yan, Jiaying Mo, Jianzhong Sheng, Hongbo Yang, Hefeng Huang

**Affiliations:** ^1^ Center for Reproductive Medicine, the Fourth Affiliated Hospital of School of Medicine, and International School of Medicine, International Institutes of Medicine Zhejiang University Yiwu China; ^2^ Institute of Medical Genetics and Development, Key Laboratory of Reproductive Genetics (Ministry of Education) and Women's Hospital Zhejiang University School of Medicine Zhejiang China; ^3^ Institute of Reproduction and Development, Shanghai Key Laboratory of Reproduction and Development, Obstetrics and Gynecology Hospital Fudan University Shanghai China; ^4^ Shanghai Key Laboratory of Female Reproductive Endocrine Related Diseases Shanghai China

**Keywords:** chromatin accessibility, exercise, gestational diabetes mellitus, offspring health, skeletal muscle

## Abstract

**Background:**

Gestational diabetes mellitus (GDM), a common pregnancy complication characterized by maternal hyperglycemia, negatively impacts offspring health. Skeletal muscle, a critical tissue for glucose and lipid metabolism, is especially vulnerable to prenatal environmental insults. However, the effects of intrauterine hyperglycemia (IUHG) on offspring skeletal muscle development remain poorly understood. This study aimed to investigate the effects of IUHG on skeletal muscle development in offspring and evaluate whether postnatal exercise could mitigate these effects.

**Methods:**

Pregnant mice were assigned to GDM and control groups. Offspring were further divided into control and exercise subgroups. Body weight, glucose tolerance test (GTT), insulin tolerance test (ITT), body composition, muscle strength and exercise capacity were assessed. At 20 weeks of age, skeletal muscle morphology was evaluated via various staining and Transmission Electron Microscope. Transcriptomic changes were analysed by RNA sequencing (RNA‐seq) and chromatin accessibility was assessed using ATAC‐seq to identify molecular mechanisms underlying IUHG‐induced alterations. Additionally, primary fetal myoblasts were cultured under normal and high‐glucose conditions to investigate metabolic changes and lipid accumulation in vitro.

**Results:**

Offspring exposed to IUHG exhibited increased body weight, impaired glucose and insulin tolerance, altered body composition, reduced muscle strength and diminished exercise capacity at adulthood. Exercise intervention in diabetic offspring improved the muscle ratio (*p* < 0.05), fat ratio (*p* < 0.05), lipid profiles (*p* < 0.005) and muscle structure and strength (*p* < 0.005). Transcriptomic and epigenomic profiling identified significant changes in genes and regulatory elements associated with immune regulation, myogenesis, lipid metabolism and inflammation in GDM‐exposed offspring. In vitro, high‐glucose exposure of E14.5d fetal myoblasts led to significant metabolic reprogramming, including lipid accumulation and disruptions in glycolysis and oxidative metabolism. Furthermore, the expression of AP‐1 family members Fos and Junb was up‐regulated in myoblasts under high‐glucose conditions, which aligns with the findings in the in vivo models.

**Conclusions:**

IUHG disrupts skeletal muscle development and metabolic function in offspring through structural, transcriptional and epigenetic alterations. Postnatal exercise partially reversed these impairments, highlighting its potential as a non‐pharmacological intervention. These findings provide new insights into the developmental origins of skeletal muscle dysfunction in GDM‐exposed offspring and underscore the importance of early prevention strategies.

## 1 Introduction

Gestational diabetes mellitus (GDM) traditionally referred to the first occurrence of abnormal maternal glucose metabolism during pregnancy, which was one of the common complications during pregnancy [1]. A growing number of studies found that maternal hyperglycemia was closely related to the occurrence of obesity, abnormal glucose and lipid metabolism, hypertension, depression and other diseases in the offspring at their adulthood [2, 3, 4, 5, 6, 7]. Skeletal muscle, as one of the important organs in regulating glucose and lipid metabolism, plays a critical role in maintaining body energy homeostasis and exercise ability [8]. Maternal obesity during pregnancy can mediate lipid deposition in skeletal muscle of offspring by affecting fetal skeletal muscle metabolism and inflammation‐related pathways [9]. However, there were only a few studies focused on the influence of gestational diabetes on the development and function of skeletal muscle in offspring [10, 11]. Studying the function of genes at the transcription level may reveal the molecular mechanism of the specific biological process of disease. Epigenetic alterations can mechanistically support skeletal muscle memory and play a pivotal role in the regulation of gene expression [12].

Epigenetic modifications play a crucial role in the abnormal transcriptional regulation of offspring skeletal muscle following intrauterine hyperglycemia (IUHG) exposure [13, 14, 15]. Chromatin accessibility studies also allow to further identify key transcription factors in the transcriptional regulation of gene expression [16]. It is unclear whether GDM exposure affects the chromatin accessibility in the skeletal muscle of offspring.

Integrated omics analysis of different time axes of GDM exposed offspring skeletal muscle to elucidate key genes, metabolites or signalling pathways causing abnormal offspring skeletal muscle development by GDM exposure. Current study can provide scientific evidence to understand the mechanism of the effect of intrauterine high glucose exposure on mammalian skeletal muscle development, which has original innovation and clinical translational significance.

## 2 Materials and Methods

### 2.1 Chemicals and Reagents

The information for all the chemicals and reagents used in this study can be found in Table S4.

### 2.2 Animal Care and Experimental Scheme

All experimental procedures were conducted in accordance with a protocol approved by the Zhejiang University Animal Experiment Center Ethics Committee (Ethics approval number: ZJU20210056). A GDM mouse model was established as previously described in our team [S1]. Female mice with vaginal plugs were designated as GD0.5 (dam) and ED0.5 (embryo). Pregnant mice were divided into control (CTR) and GDM groups. GDM mice received streptozotocin (STZ, 100 mg/kg) dissolved in citrate buffer by intraperitoneal injection on GD6.5 and GD12.5 after overnight fasting; CTR mice received buffer only. Blood glucose was measured daily from GD13.5 via tail vein sampling, with GDM defined as random glucose > 16.8 mmol/L. The detailed animal modelling protocol and offspring grouping can be found in Supporting Information S1: Methods S1.

### 2.3 Exercise Protocol

The detailed offspring exercise protocol can be found in Supporting Information S1: Methods S2.

### 2.4 Weight Monitoring and Body Composition Analysis

Body weight of offspring mice was recorded at fixed times from birth until sacrifice. Body composition was assessed using the QMR06‐090H Low‐Field Magnetic Resonance Analyser (Suzhou NIUMAG ANALYTICAL INSTRUMENT CORPORATION, Suzhou, Jiangsu). Mice were weighed, identified and placed on the instrument for measurements using a 60 mm probe coil at a resonance frequency of 21.768 MHz.

### 2.5 Glucose Tolerance Test (GTT) and Insulin Tolerance Test (ITT)

At 8, 12, 16 and 20 weeks old, offspring mice fasted overnight or for 16 h. After measuring body weight, glucose (2 g/kg) or insulin (0.8 IU/kg) was administered intraperitoneally (i.p.) into the abdomen and blood glucose was measured at multiple time points (0, 30, 60 and 120 min) post‐injection. The area under the curve (AUC) was calculated.

### 2.6 Biochemistry Assay

The levels of mouse serum triglycerides (TG), total cholesterol (TC), high‐density lipoprotein (HDL) and low‐density lipoprotein (LDL) were analysed using the Toshiba TBA‐120FR fully automated biochemical analyser. Blood samples were acquired as above mentioned. The supernatant was collected for subsequent insulin level testing. Mouse insulin levels were measured using a Mouse Ultra‐Sensitive Insulin ELISA Kit (Crystal Chem Insulin ELISA Kit, United States, CAT NO 90095).

### 2.7 Grip Strength Testing

The Grip Strength Meter (XR 501, Shanghai Xinruan) was used to test skeletal muscle strength in mice. Mice were allowed to adapt to the test environment for 30 min. After preparation, they were pulled back evenly until their limbs left the plate. The average of three measurements was recorded.

### 2.8 Histology Examination in Skeletal Muscle of Offspring

Including the experiment of HE staining, Oil Red O Staining, Nile Red staining and TEM scanning. All of the experiments were performed as described in Supporting Information S1: Methods S3.

### 2.9 Detection of Triglyceride (TG) Content in Skeletal Muscle

The detailed protocol can be found in Supporting Information S1: Methods S4.

### 2.10 Preparation of Skeletal Muscle Single Cell Suspension

Chop skeletal muscle tissue, transfer to a 15 mL centrifuge tube, add 7 mL MDB solution and incubate in a 37°C shaking water bath for 1 h. Add WM solution to 15 mL, centrifuge at 750 g for 5 min, then add collagenase II and dispase. Incubate at 37°C for 30 min, centrifuge and resuspend the pellet. Filter through a 40 μm nylon cell strainer, then resuspend the pellet in WM solution for cell counting and further experiments. To determine the purity of muscle cells in the single‐cell suspension, we used single‐cell sequencing technology to assist in cell composition analysis and quantification. The single‐cell experimental protocol can be found in Supporting Information S1: Methods S5.

### 2.11 Transcriptional Analysis of Mice Skeletal Muscle

Transcriptional sequencing and analysis were performed as described in Supporting Information S1: Methods S6.

### 2.12 Chromatin Accessibility Analysis of Mice Skeletal Muscle

Chromatin accessibility analysis and analysis was performed as described in Supporting Information S1: Methods S7.

### 2.13 Bioinformatics Analyses

Transcriptomic data were processed using cutadapt‐1.9 to remove adapters and filter low‐quality sequences. Clean data were aligned to the GRCm38/mm10 genome using hisat2–2.0.4 and transcript assembly was performed with stringtie‐1.3.4d. Differential expression was analysed using DESeq2, with genes having a fold change > 2 or < 0.5 and *p*‐value < 0.05. GO and KEGG enrichment analyses followed. For ATAC‐seq, BED Tools and annotate Peaks were used to analyse read counts overlapping genomic features. Differential accessibility was assessed with DESeq2 and a heatmap of promoter enrichment was generated using R. Functional enrichment analysis was performed using the ggplot2 package in R (version 4.2.1) for visualization and Gene Set Enrichment Analysis (GSEA) was conducted using the cluster Profiler package.

The Expanded Research Design and Methods in Supporting Information S1: Methods S8–S12 include detailed descriptions of the following: quantitative Real‐time PCR, Western Blotting, extraction of mouse fetal primary myoblasts and high‐glucose intervention, untargeted metabolomics, Nile Red staining.

### 2.14 Statistical Analysis

The results were presented as mean±standard error of the mean (S.E.M). The statistics were analysed by one‐way ANOVA and two‐way ANOVA. A *p*‐value < 0.05 was considered statistically significant. Prism version 8.0 (GraphPad Software Inc., San Diego, CA, USA) was used for statistical analysis.

## 3 Results

### 3.1 Impact of IUHG on Growth Development and Skeletal Muscle Function of Offspring

#### 3.1.1 Body Weight Feature and Body Composition Analysis of Offspring

**FIGURE 1:**
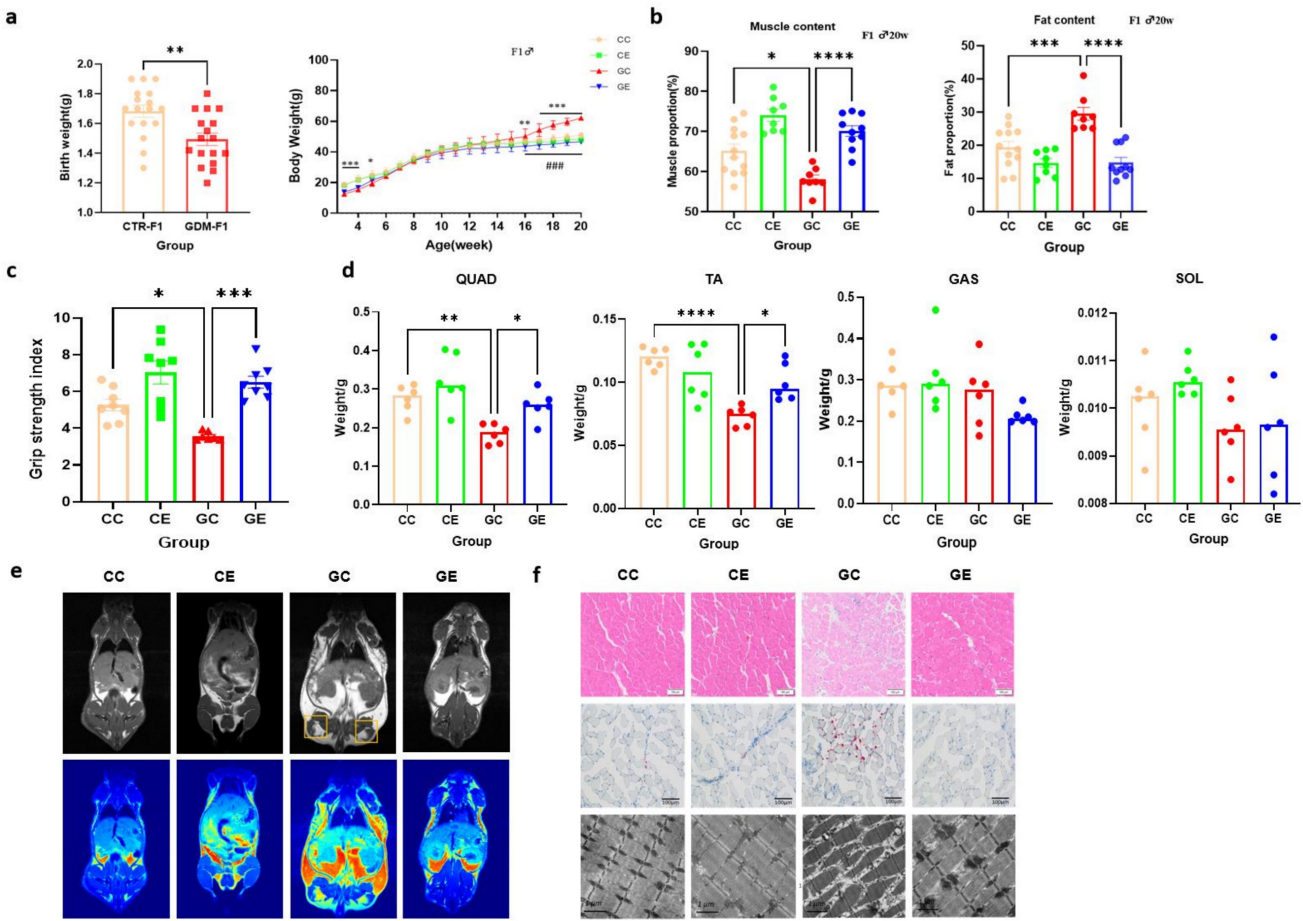
Impact of IUHG on Growth Development and Skeletal Muscle Function of Offspring. (a) Birth weight of offspring and body weight curve of male mice offspring, for birth weight:GDM‐F1 (*n* = 17 pups, from 6 litters) vs. CTR‐F1(*n* = 17 pups, from 6 litters) **p < 0.01, using Student's *t*‐test, data are expressed as Mean ± SEM. For body weight curve: GC vs. CC *p < 0.05, **p < 0.01, ***p < 0.005; GE vs. GC ###*p* < 0.005; One‐way ANOVA test was used and the data are expressed as Mean ± SEM, *n* = 8–12/group (every group from 6 litters); (b) Proportion of muscle and fat content in offspring of male mice, *p < 0.05, ***p < 0.005, ****p < 0.001. One‐way ANOVA test was used and data were expressed as Mean ± SEM, *n* = 8–12/group (every group from 6 litters); (c) Skeletal Muscle grip strength index test results of 20‐week‐old male offspring, *n* = 8/group (every group from 4 litters); (d) Different type of skeletal muscle mass in 20‐week‐old male offspring, *n* = 6/group (every group from 3 litters); (e) MRI scanning image for fat test of 20‐week‐old male offspring, white part marked by yellow square represents fat accumulation in hindlimb, *n* = 3/group (every group from 3 litters); (f) Skeletal Muscle morphology examination of 20‐week‐old male offspring, separately represent HE staining (10×), Oil Red (20×), TEM (5900×), *n* = 4/group (every group from 4 litters).

Offspring from GDM exhibited significantly reduced birth weights compared to the CTR group (Figure 1a). Subsequent weight monitoring showed that before 3 weeks old, GDM offspring weighed less than their CTR counterparts (Figure 1a). Post‐weaning, the GDM group surpassed the CTR group in weight at 16 weeks old (Figure 1a). Post‐weaning exercise prevented weight gain in GDM male offspring from 17 weeks old (Figure 1a). At 20 weeks old, GDM male offspring showed increased body fat and reduced muscle mass compared to CTR male offspring (Figure 1b). Exercise reversed these changes, reducing fat and increasing muscle proportions (Figure 1b).

#### 3.1.2 Muscle Strength and Mass Assessment in Adult Offspring Mice

Muscle strength test results revealed a significant decrease in the relative grip strength index in the GDM offspring group, whereas the GDM offspring exercise group displayed a significantly higher grip strength index compared to the GDM offspring group (Figure 1c). This indicates a decline in muscle strength in male offspring of GDM, while treadmill exercise appears to mitigate this adverse skeletal muscle behavioural phenotype in GDM male offspring.

At 20 weeks old, the mass of the quadriceps (QUAD) and the tibialis anterior (TA) muscle in the GDM male mice was found to be lighter than those in the CTR group. Exercise was able to alleviate the reduction in muscle mass for both types of skeletal muscles observed in the GDM offspring group. However, no significant differences were found in the mass of the gastrocnemius (GAS) and soleus (Sol) muscles among different groups (Figure 1d).

#### 3.1.3 Skeletal Muscle TG Content and Lipid Accumulation in Adult Male Offspring

In the skeletal muscle tissue of 20‐week‐old male offspring mice, TG was increased in the GC group compared with the CC group, while TG in the GE group was significantly lower than that in the GC group (Figure S1). Similarly, through small animal Magnetic Resonance Imaging (MRI) scanning examinations, we found that the offspring of GDM mice exhibited localized lipid accumulation in skeletal muscles throughout the body and in the lower limbs at 20 weeks of age, which can be alleviated via long term treadmill exercise (Figure 1e).

#### 3.1.4 Skeletal Muscle Morphology Phenotype of Adult Male Offspring

HE staining showed a significant reduction in the cross‐sectional area of the tibial anterior muscle fibres in the 20‐week GDM group compared to the CTR offspring (*p* < 0.005) and it increased in the GDM exercise group (*p* < 0.05) (Figures 1f and S2a). In the skeletal muscle oil red O staining, we observed more lipid droplets in the GC group compared to the control (*p* < 0.001), while exercise significantly reduce the number of lipid droplet in the skeletal muscle (*p* < 0.001) (Figures 1f and S2b). TEM results showed obviously disorganized arrangement of myofibrillar structures in the skeletal muscle of GC group, with swelling and vacuoles appeared in mitochondria (Figure 1f), the percentage of impaired mitochondria in GC group is significantly more than the CC group (Figures 1f and S2c). Compared with the GC group, the skeletal muscle and filaments of the GE group were neatly arranged, the mitochondrial structure outline was still recognizable and some mitochondrial shape was slightly enlarged, the percentage of impaired mitochondria in GE group is significantly lower than the CC group (Figures 1f and S2c).

### 3.2 Metabolic Function in Adult Male Offspring Exposed to IUHG

#### 3.2.1 Glucose Tolerance Tests (GTTs)and Insulin Tolerance Tests (ITT)

At 8 weeks old, GDM male offspring showed worse glucose tolerance than control offspring, which can be improved via aerobic exercise (Figure S3a). By 12 weeks old, differences in glucose tolerance showed persistent abnormalities, while exercise can continue to mitigate these effects (Figure S3b). No significant differences were noted in insulin tolerance at 8 and 12 week‐old offspring. From 16 weeks old, GDM male offspring exhibited significant insulin resistance, which can be rescued by aerobic exercise (Figure S3c,d).

#### 3.2.2 Lipid Metabolism

At 20 weeks old, GDM male offspring had elevated TC, TG, LDL and NEFA levels compared to controls. Exercise significantly reduced the level of TC, TG and LDL in GDM male offspring (Table 1).

**TABLE 1:** Serum lipid metabolism markers level.

	CC (*n* = 8)	CE (*n* = 8)	GC (*n* = 8)	GE (*n* = 8)
TC(mmol/L)	2.74 ± 0.47	2.76 ± 0.43	3.91 ± 1.04**	2.99 ± 0.42#
TG(mmol/L)	1.98 ± 0.44	1.81 ± 0.39	2.96 ± 0.66**	2.30 ± 0.37#
LDL(mmol/L)	0.89 ± 0.06	0.85 ± 0.02	1.10 ± 0.22**	0.91 ± 0.07#
HDL(mmol/L)	2.45 ± 0.51	2.81 ± 0.45	2.11 ± 0.47	2.51 ± 0.58
NEFA(mmol/L)	2.24 ± 0.57	2.49 ± 0.51	3.33 ± 1.17*	2.66 ± 0.48

*Note:* *N* = 8/group (Each group consists of mice from four different litters, with two mice randomly selected from each litter), CC vs. GC *p < 0.05, **p < 0.01, GC vs. GE #*p* < 0.05. One‐way ANOVA test was used, and data were expressed as Mean ± SEM.

### 3.3 Impact of GDM on Skeletal Muscle Transcriptomics at Different Developmental Stages in Offspring

#### 3.3.1 Functional Enrichment Results of Differential Genes in Skeletal Muscles of Different Periods of Offspring

**FIGURE 2:**
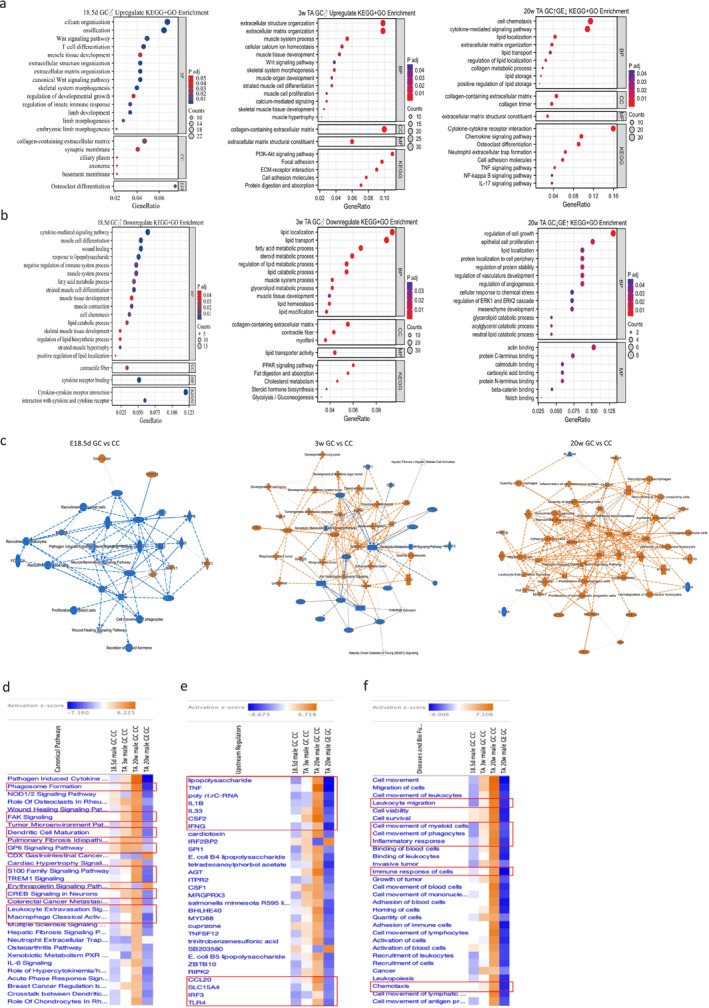
Transcriptomics at Different Developmental Stages in skeletal muscle of Offspring. (a) Up‐regulated Gene Ontology (GO) terms and Kyoto Encyclopedia of Genes and Genomes (KEGG) pathway enrichment analysis of offspring skeletal muscle after intrauterine hyperglycemia exposure in transcriptional level; (b) Down‐regulated Gene Ontology (GO) terms and Kyoto Encyclopedia of Genes and Genomes (KEGG) pathway enrichment analysis of offspring skeletal muscle after intrauterine hyperglycemia exposure in transcriptional level; (c) Graphical Summary analysis of IPA analysis of offspring skeletal muscle after intrauterine hyperglycemia exposure in transcriptional level at different development stages; (d) Canonical Pathways dynamic analysis of offspring skeletal muscle feature from E18.5d to 20 weeks old, GC compared to CC group and GE compared to GC group; (e) upstream regulators of offspring skeletal muscle variation from E18.5d to 20 weeks old, GC compared to CC group and GE compared to GC group; (f) Diseases and Bio Functions of offspring skeletal muscle feature from E18.5d to 20 weeks old, GC compared to CC group and GE compared to GC group. Each group contains 3 biological replicates. For the E18.5d group, each biological replicate is composed of all foetuses from 3 independent litters (total of 9 litters). The number of foetuses in each biological replicate for the control group (CTR‐F1) was 7, 8 and 10, respectively, and for the GDM group (GDM‐F1) was 4, 5 and 5, respectively. For the 3‐week‐old and 20‐week‐old groups, each biological replicate consists of one sample from a single mouse, with each mouse coming from a different litter.

Functional enrichment analysis of up‐regulated differential genes in E18.5d GDM male offspring skeletal muscle showed enrichment in ciliogenesis, Wnt signalling, T‐cell differentiation and muscle development processes (Figure 2a). In 3‐week‐old offspring, upregulated genes were enriched in extracellular matrix organization, muscle formation, calcium ion homeostasis and the PI3K‐Akt pathway (Figure 2a). At 20 weeks, genes upregulated in GDM and downregulated with exercise enriched in chemotaxis, cytokine signalling, lipid transport and NF‐κB pathways (Figure 2a). Down‐regulated genes in E18.5d GDM offspring were enriched in cytokine signalling, myocyte development, immune regulation and muscle system development (Figure 2b). In 3‐week‐old GDM offspring, downregulated genes enriched in lipid metabolism, PPAR signalling and cholesterol biosynthesis (Figure 2b). At 20 weeks, genes downregulated in GDM and upregulated with exercise enriched in cell growth regulation and lipid metabolism (Figure 2b). Compared to the CC group, the pathways up‐regulated in the ce group are mainly skeletal muscle contraction, muscle system process and T cell mediated immunity, while the pathways downregulated are mainly cell chemotaxis, regulation of inflammatory response and lipid localization (Figure S5a). At E18.5d, GSEA analysis of GDM offspring skeletal muscle showed suppressed defence and inflammatory pathways (negative NES) and active microtubule formation (positive NES), indicating immune suppression (Figure 6a). In 3 weeks old, inflammatory pathways were activated (positive NES), while lipid catabolism was down‐regulated (negative NES) (Figure 6b). At 20 weeks, the GC group showed active inflammation (positive NES) (Figure 6c), whereas the GE group had reduced inflammation (negative NES) (Figure 6d), suggesting exercise mitigates chronic inflammation. Compared with CC group, ce group showed regulation of the striated muscle tissue development (positive NES), adaptive immune response (positive NES), suggesting exercise in control group enhance skeletal muscle funcion and immune ability (Figure S5b).

#### 3.3.2 IPA Analysis of Offspring Skeletal Muscle at Different Developmental Stages

In E18.5d male GDM offspring, immune and inflammatory response molecules (IRF3, TLR3, IL1A, MYD88) and pathways were suppressed. At 3 weeks, oncogenes and kinases (ERBB2, MAPK1, FOXM1) were activated. At 20 weeks, immune‐related molecules (NF‐κB, ICAM1, TNF) were activated, but the Th2 pathway and IL15RA were suppressed. In the exercise group, immune suppression (SAMD11, DOCK8) and reduced inflammatory pathways were observed (Figure 2c).

A color gradient from blue to red indicates the activation status of pathways, with blue representing suppressive (negative Z‐score) effect and red indicating activated (positive Z‐score) effect. The results from the Canonical Pathways analysis reveal that pathways such as the Pathogen Induced Cytokine Storm Signalling Pathway, Phagosome Formation, FAK Signalling, Dendritic Cell Maturation, GP6 Signalling Pathway, S100 Family Signalling Pathway, TREM1 Signalling and CREB Signalling in Neurons progressively exhibit activation states in the skeletal muscles of male offspring exposed to high glucose in utero, whereas they are suppressed in the exercise group exposed to in utero high glucose (Figure 2d). The dynamic changes in Upstream Regulators indicate that molecules such as lipopolysaccharide (LPS), TNF, poly rI:rC‐RNA, IL1B, IL33, CSF2, IFNG, CCL20, SLC15A4, IRF3 and TLR4 progressively show activation states in the skeletal muscle of male offspring exposed to intrauterine hyperglycemia, while they were suppressed in the exercise group (Figure 2e). The analysis of Diseases and Bio Functions showed that biological functions such as leukocyte migration, immune response and chemotaxis also progressively became activated in the skeletal muscles of male offspring exposed to intrauterine hyperglycemia, yet these functions were suppressed in the exercise group (Figure 2f).

#### 3.3.3 qPCR Validation of Transcriptomics Results

In conjunction with functional enrichment analyses, we conducted qPCR validation for key genes implicated in inflammatory immune responses and chemotactic activities. Specifically, we validated eight immune response‐related genes (TLR1, TLR2, TLR8, TLR13, Cd80, Cd28, Cd86, Itgb7), which predominantly showed upregulation in the Gestational Control (GC) group and downregulation in the Gestational Exercise (GE) group, except for Cd80 which did not exhibit reversal in the GE group (Figure S7a). For inflammatory response, five genes (Il1a, Il1b, Il10, Ly86, Nlrp3) were upregulated in the GC group and downregulated in the GE group (Figure S7b). Lastly, among chemotactic activity genes, nine genes (Cxcl3, Cxcl5, Cxcr6, Ccl3, Ccl4, Ccl6, Ccl9, Ccl12, Ccl20) were validated, which were upregulated in the GC group and downregulated in the GE group (Figure S7c). This qPCR validation supported the differential expression patterns suggested by our enrichment analysis, confirming the impact of IUHG exposure on gene expression related to immune and inflammatory responses and chemotactic activities.

### 3.4 Impact of GDM on Chromatin Accessibility Changes in Offspring at Different Developmental Stages

#### 3.4.1 Distribution of Genome Peaks in Offspring Mice Skeletal Muscle

Analysis of chromatin peaks in offspring skeletal muscle at various developmental stages revealed that most peaks were located in intronic regions and distal intergenic spaces, accounting for over two‐thirds of all peaks (Figure S8). Peaks within promoter regions constituted 8.4%–12.1%, while exonic regions had 4.4%–5.5%. A decline in peak numbers was noted in GDM offspring compared to controls, with a progressive reduction with age. At 20 weeks, the GE group displayed a higher number of enriched peaks than the GC group, indicating that long‐term exercise might enhance chromatin openness (Figure S8a).

#### 3.4.2 Heatmap of Chromatin Open Regions Peaks in Offspring Mice Skeletal Muscle

**FIGURE 3:**
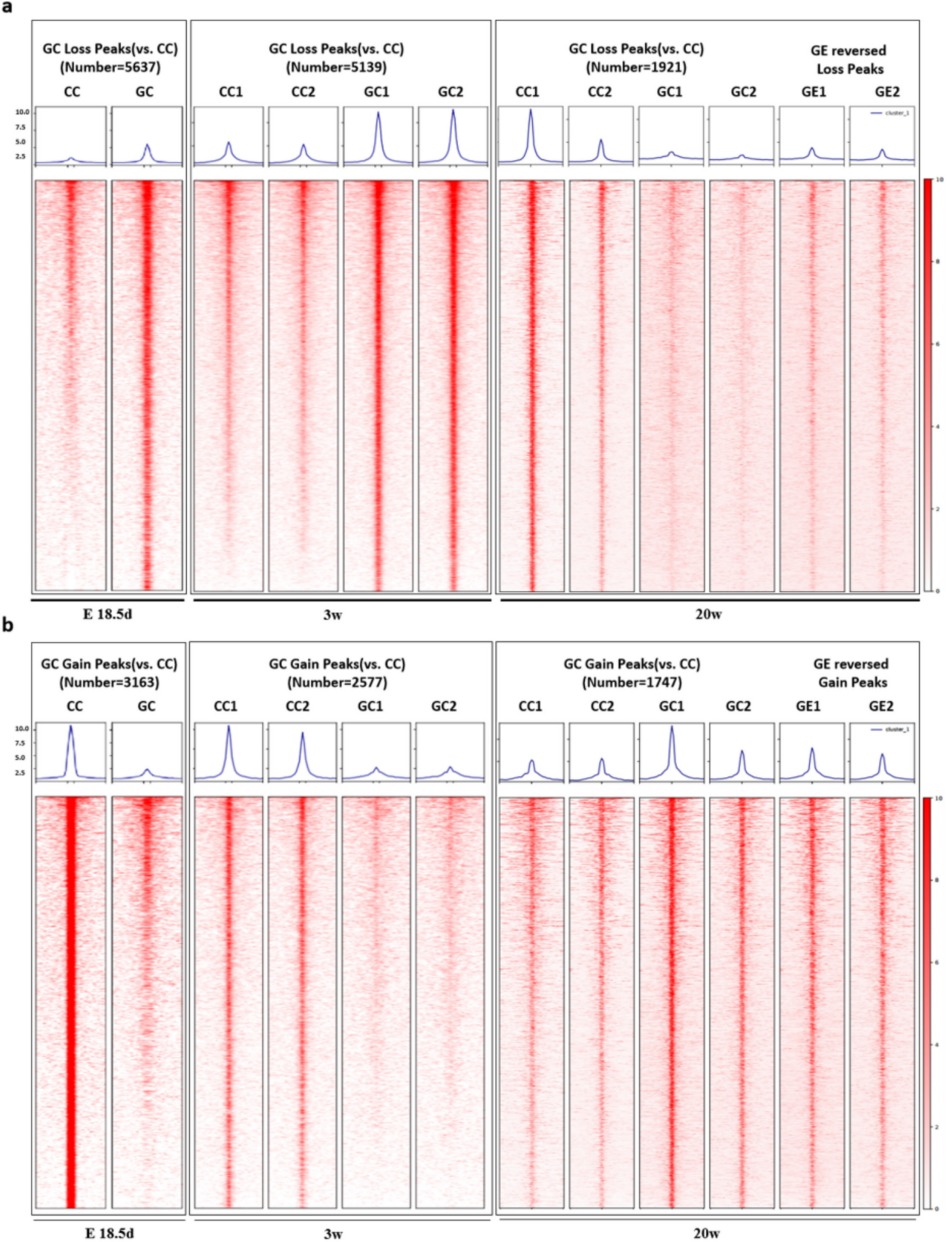
Heatmap of the peak signals across the gene body of library; ±2.0 represents upstream and downstream of the TSS. (a) Loss peaks heatmap of offspring skeletal muscle after intrauterine hyperglycemia exposure at E18.5d, 3 weeks old, 20 weeks old as well as exercise reversed group; (b) Gain peaks heatmap of offspring skeletal muscle after intrauterine hyperglycemia exposure at E18.5d, 3 weeks old, 20 weeks old as well as exercise reversed group. For E18.5d, the CTR‐F1 group consisted of 25 foetuses from 3 litters and the GDM‐F1 group consisted of 26 foetuses from 5 litters. Each biological replicate was composed of mixed samples from different litters (1:1 comparison). For the 3‐week‐old and 20‐week‐old groups, each biological replicate consisted of one mouse from a different litter, with each group having two biological replicates (2:2 comparison).

In E18.5d GDM offspring skeletal muscle, 5637 accessible peaks were lost (Figure 3a) and 3163 peaks were gained, indicating a net reduction in chromatin accessibility (Figure 3b). A similar decrease in chromatin openness was observed in 3‐week‐old GDM offspring, with 5139 peaks lost (Figure 3a) and 2577 gained (Figure 3b). At 20 weeks, the GDM offspring had 1921 lost (Figure 3a) and 1747 gained (Figure 3b) peaks, showing reduced chromatin accessibility. In contrast, the GE group reversed gained more peaks (3104) than lost (980) peaks (Figure 3a,b), indicating increased chromatin openness in the skeletal muscle of 20‐week‐old GE offspring.

#### 3.4.3 Functional Enrichment Analysis of Chromatin Accessibility Regions in Skeletal Muscle of Offspring

**FIGURE 4:**
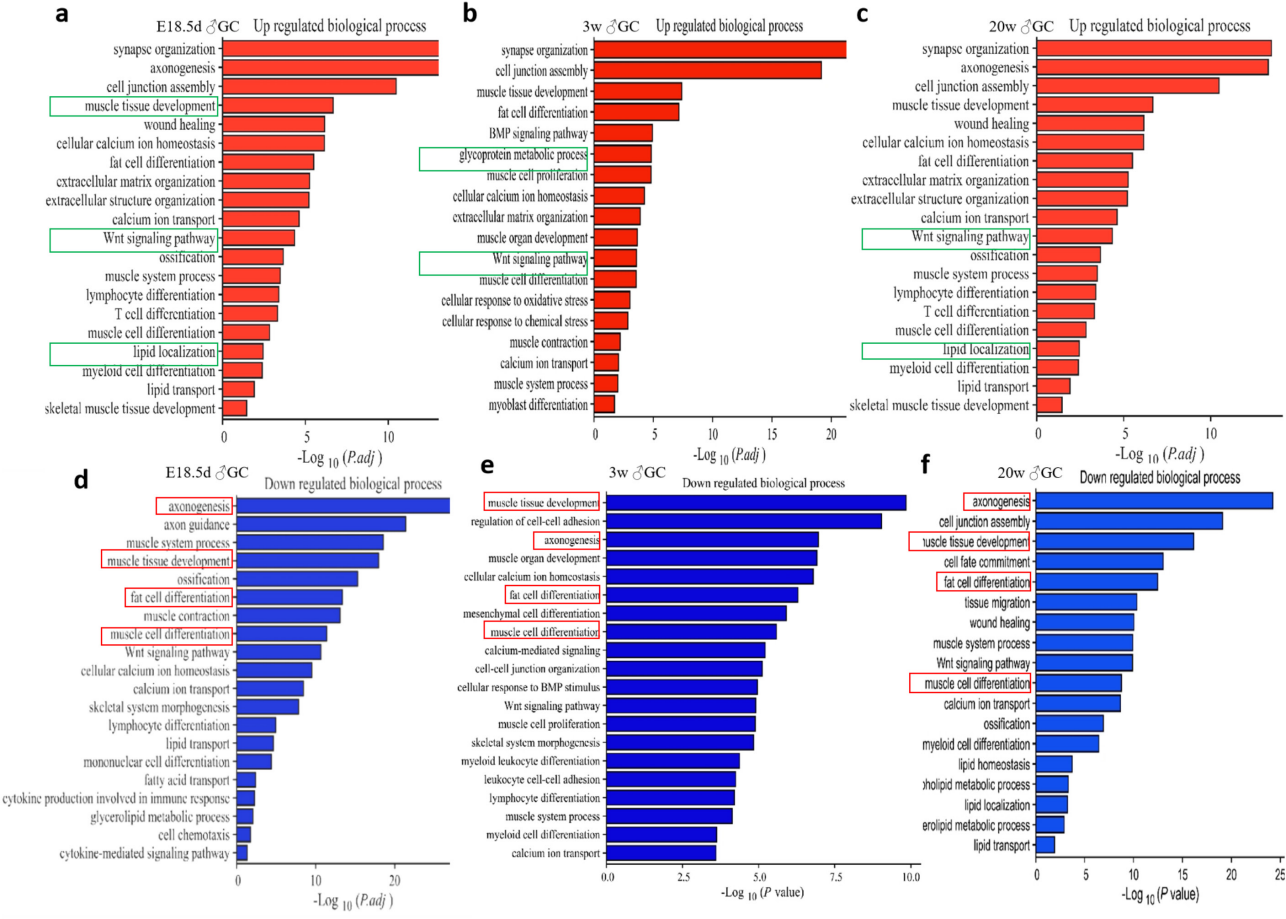
Gene Ontology (GO) terms analysis of offspring skeletal muscle after intrauterine hyperglycemia exposure in chromatin accessibility level. (a–c) Up‐regulated terms and pathway in GDM male offspring skeletal muscle from E18.5d to 20 weeks old; (d–f) Down‐regulated terms and pathway in GDM male offspring skeletal muscle from E18.5d to 20 weeks old. For E18.5d, the CTR‐F1 group consisted of 25 foetuses from 3 litters and the GDM‐F1 group consisted of 26 foetuses from 5 litters. Each biological replicate was composed of mixed samples from different litters (1:1 comparison). For the 3‐week‐old and 20‐week‐old groups, each biological replicate consisted of one mouse from a different litter, with each group having two biological replicates (2:2 comparison).

In E18.5d GDM offspring skeletal muscle, KEGG analysis revealed increased chromatin accessibility in processes such as calcium ion homeostasis, lipid transport and T‐cell activation. At 3 weeks, increased accessibility involved cell junction assembly, fat cell differentiation and myoblast differentiation. In 20‐week‐old offspring, up‐regulated pathways included MAPK, Hippo, Wnt, insulin secretion and extracellular matrix interactions. Down‐regulated accessibility in E18.5d GDM offspring affected axonogenesis, muscle development and fatty acid oxidation. In 3‐week‐old offspring, processes like muscle development, mesenchymal stem cell differentiation and lymphocyte differentiation were down‐regulated (Figure 4).

#### 3.4.4 Motif Analysis of Differential Peaks in the Skeletal Muscle of Offspring Mice

**FIGURE 5:**
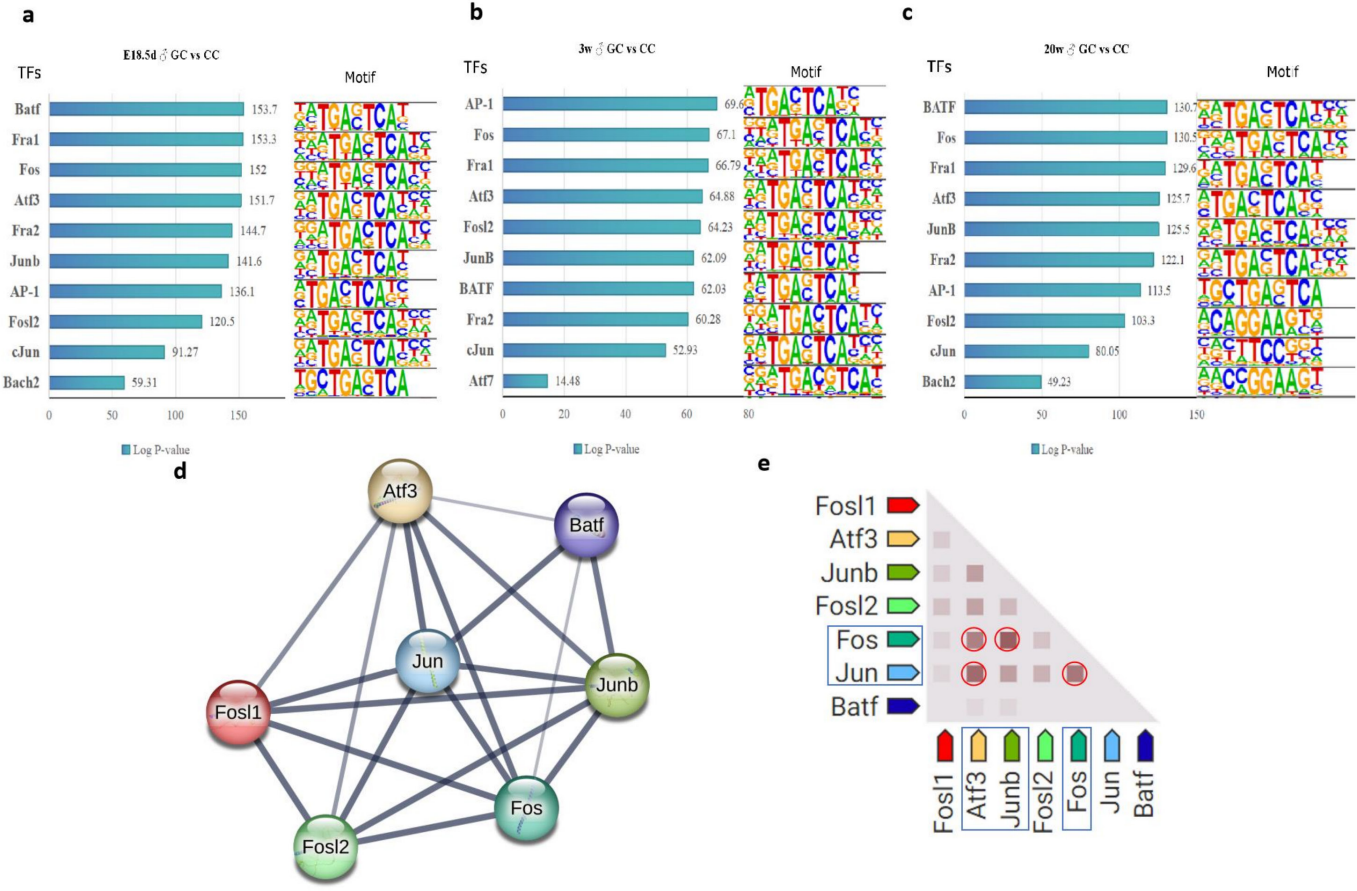
Motifs analysis of genes associated with differential chromatin accessibility and STRING analysis of differential chromatin accessibility binding motifs transcription factor. (a) Enriched transcription factor binding motifs in skeletal muscle of E18.5d male offspring (GC vs. CC); (b) Enriched transcription factor binding motifs in skeletal muscle of 3‐week‐old male offspring (GC vs. CC); (c) Enriched transcription factor binding motifs in skeletal muscle of 20‐week‐old male offspring (GC vs. CC); (d) The protein–protein interaction network among AP‐1 family members; (e) A matrix of interactions between different AP‐1 family members, with red circles highlighting significant interactions. For E18.5d, the CTR‐F1 group consisted of 25 foetuses from 3 litters and the GDM‐F1 group consisted of 26 foetuses from 5 litters. Each biological replicate was composed of mixed samples from different litters (1:1 comparison). For the 3‐week‐old and 20‐week‐old groups, each biological replicate consisted of one mouse from a different litter, with each group having two biological replicates (2:2 comparison).

We identified significant enrichment of motifs associated with differential transcription factors in chromatin accessible regions of skeletal muscle in GDM male offspring across different developmental stages. At E18.5d, enriched transcription factors included BATF, Fra1, Fos, Atf3, Fra2, JunB, AP‐1, Fosl2, Jun‐AP1 and Bach2, all sharing the palindrome sequence‐TGATCA‐(Figure 5a). At 3 weeks, AP‐1, Fos, Fra1, Atf3, Fosl2, JunB, BATF, Fra2 and Atf7 showed similar enrichment (Figure 5b). At 20 weeks, BATF, Fos, Fra1, Atf3, JunB, Fra2, AP‐1, Fosl2, cJun and Bach2 were enriched in chromatin accessible regions (Figure 5c). To identify key transcription factors, we analysed motif overlap using the STRING database, revealing predominant interactions within the AP‐1 family. The network diagram showed strong connections between Atf3, BATF, Jun, JunB, Fos, Fosl1 and Fosl2 (Figure 5d). The interaction matrix highlighted strong AP‐1 family interactions, especially between Fos and Jun proteins, indicating their critical role in skeletal muscle development and response to intrauterine diabetic conditions (Figure 5e).

### 3.5 Western Blot Analysis Expression of Fos and Junb in Skeletal Muscle

**FIGURE 6:**
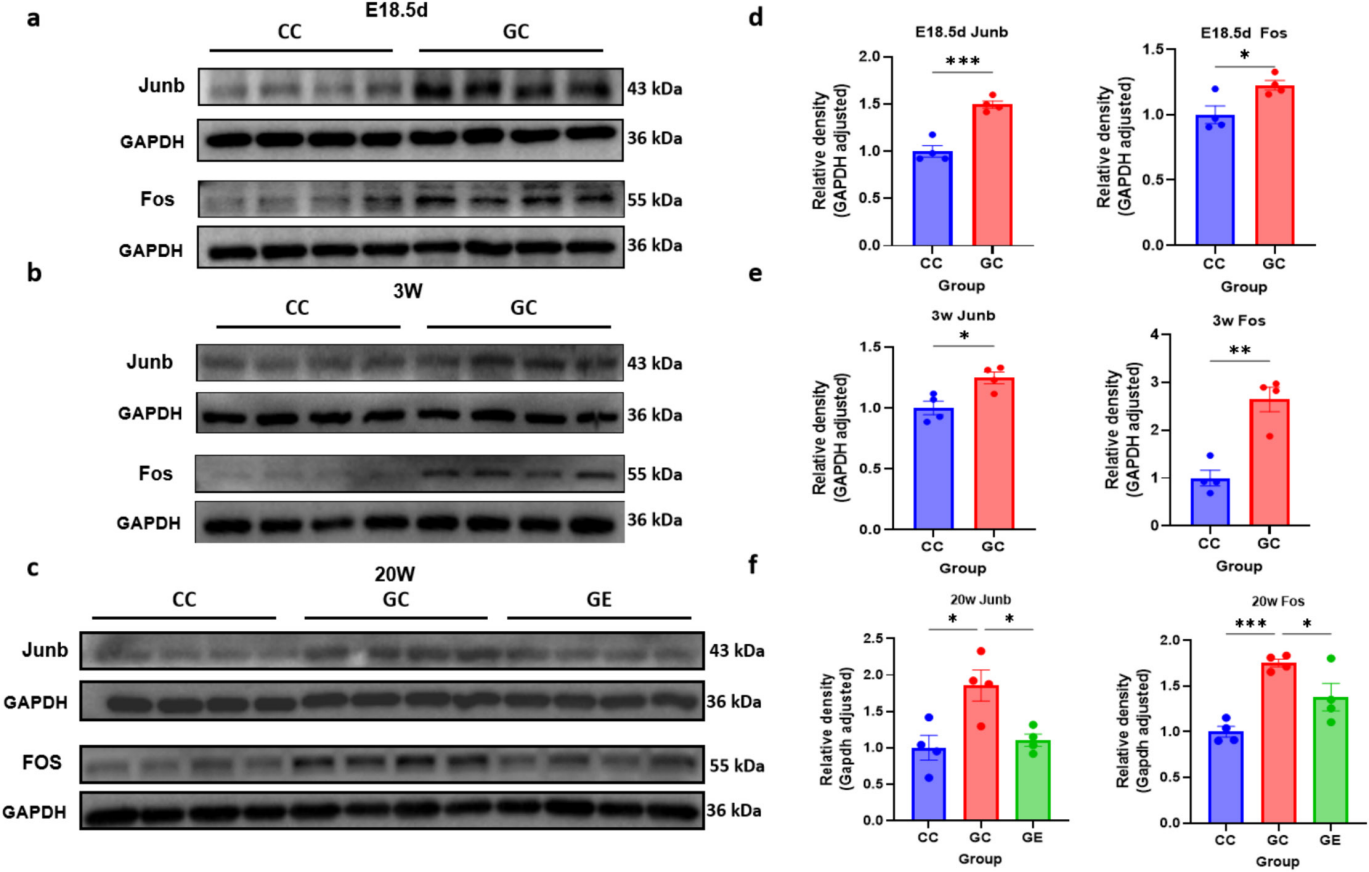
Western blot analysis of Junb and Fos expression in skeletal muscle at different developmental stages. (a, d) E18.5d, (b, e) 3 weeks and (c, f) 20 weeks. *n* = 4/group (every group from 4 litters). Protein levels of Junb and Fos were examined in skeletal muscle tissue from the control (CC), GDM (GC) and exercise (GE) groups. GAPDH was used as an internal control. Quantification of relative protein density (adjusted for GAPDH) for Junb and Fos in each group at the respective time points is shown on the right.

Western blot analysis showed that Junb and Fos protein expression were significantly increased in GDM offspring (GC) compared to control offspring (CC) at all developmental stages (E18.5d, 3 week old and 20 week old) (Figure 6). Specifically, at E18.5d, Junb and Fos levels were both significantly higher in GDM offspring (*p* < 0.005 for Junb, *p* < 0.05 for Fos) (Figure 6a,d). At 3 weeks, the reduction in Junb and Fos expression persisted at higher levels (*p* < 0.05 for Junb, *p* < 0.01 for Fos) (Figure 6b,e). At 20 weeks, Junb expression remained significantly higher in GDM offspring (*p* < 0.05), as well as Fos levels were also higher than CC group (*p* < 0.005) (Figure 6c,f). Exercise intervention (GE) at 20 weeks significantly restored Junb and Fos levels in the GDM offspring (*p* < 0.05 for both) (Figure 6c,f).

### 3.6 Effects of In Vitro High Glucose Exposure on the Metabolic Microenvironment of Fetal Myoblasts

**FIGURE 7:**
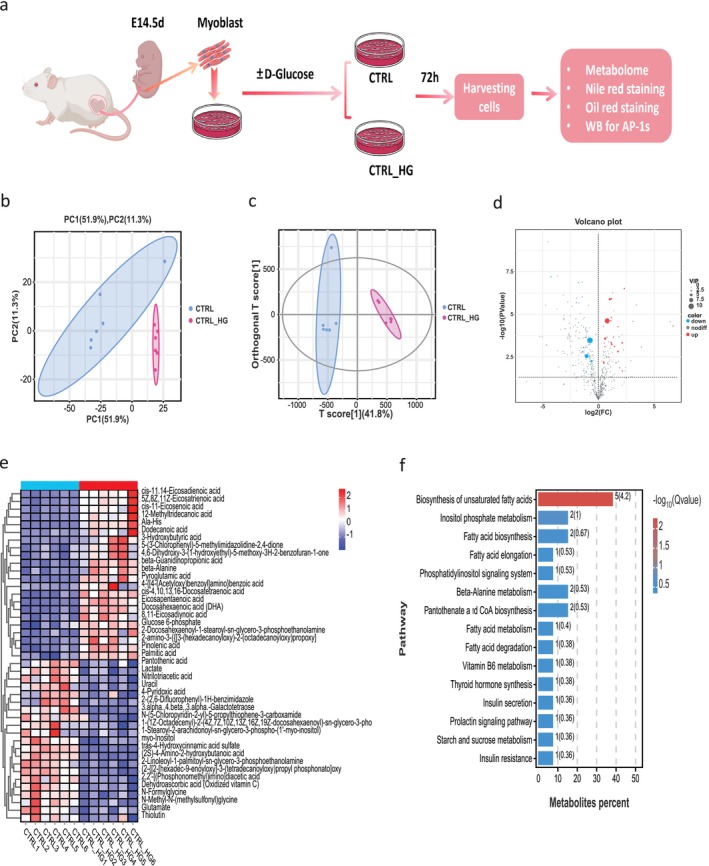
In Vitro High‐Glucose Intervention on the Metabolic Microenvironment of Fetal Mouse Myoblasts. (a) Experimental workflow showing the process of high‐glucose intervention on fetal mouse myoblasts, with subsequent analyses including metabolomics, Nile Red staining, Oil Red O staining and Western blot (WB) for AP‐1 family proteins; (b, c) Principal Component Analysis (PCA) and Orthogonal Partial Least Squares Discriminant Analysis (OPLS‐DA) score plots illustrating the distinct metabolic profiles between control (CTRL) and high‐glucose (CTRL_HG) groups; (d) Volcano plot highlighting the significant metabolites altered in response to high‐glucose exposure, with upregulated metabolites in red and downregulated metabolites in blue; (e) Heatmap showing the enrichment of differentially expressed metabolites, emphasizing the key metabolic changes; (f) KEGG pathway enrichment analysis revealing the major biological processes impacted by high‐glucose exposure. *N* = 6/group (every group from 6 litters, each litter of pups, from which myoblasts are extracted, constitutes one biological replicate).

To further investigate the direct effects of high glucose exposure on skeletal muscle, primary myoblasts were isolated from E14.5 mouse fetal skeletal muscle and cultured under control (CTRL) or high‐glucose (CTRL_HG) conditions for 72 h. Metabolomic analysis revealed distinct metabolic changes between the CTRL and CTRL_HG groups. PCA and OPLS‐DA (Figure 7a,b) confirmed the significant metabolic shift, with a clear separation between the two groups, suggesting that high glucose exposure induces substantial metabolic disruptions. Based on the variable importance on projection (VIP) > 1.0 and *p* < 0.05, we identified 43 significantly altered metabolites (Figure 7c,d). A heatmap of the comparative analysis exhibited the expression levels of the metabolites (Figure 7e). Compared with the CTRL, high glucose exposure (CTRL_HG) significantly increased the metabolites content of Pyroglutamic acid, Palmitic acid, Glucose 6‐phosphate, Pinolenic acid, while the contents of Glutamate, myo‐Inositol, 4‐Pyridoxic acid, Nitrilotriacetic acid were decreased in the HG group.

Metabolomic profiling highlighted significant changes in the lipid metabolism pathway involved in fatty acid biosynthesis and elongation (Figure 7f). The increased accumulation of lipid droplets in myoblasts exposed to high glucose was evident from Nile Red and Oil Red O staining (Figure S9a). Quantification of these staining results showed a significant increase in lipid accumulation in the CTRL_HG group compared to controls (***p < 0.005 for Nile Red fluorescence intensity, ****p < 0.001 for Oil Red O area) (Figure S9c,d). Additionally, Western blot analysis (Figure S9b) revealed that high glucose exposure significantly increased the expression of the AP‐1 family transcription factors Junb and Fos in myoblasts (**p < 0.01 for Junb, **p < 0.01 for Fos) (Figure S9e,f), which is consistent with the results we observed in the in vivo animal model.

## 4 Discussion

The incidence of GDM has been increasing globally, drawing attention to its impact on offspring health, particularly from high glucose exposure during pregnancy [17]. Pregnancy is a critical period for skeletal muscle development [18]. Our study found that IUHG led to metabolic dysfunction and abnormal skeletal muscle phenotype in adult offspring, though exercise mitigated these adverse effects. Multi‐omics analyses revealed that GDM exposure alters immune‐inflammatory responses in offspring skeletal muscle across developmental stages, with exercise alleviating these phenotypes by inhibiting inflammation‐related pathways.

Skeletal muscle has a lower priority for nutrient allocation during intrauterine development stage; therefore, it was particularly vulnerable to adverse intrauterine exposure [19, 20]. Previous studies showed that poor fetal conditions can impair skeletal muscle development and function in adulthood [21, 22]. Our study found that GDM‐exposed offspring had low birth weight, with a trend toward catch‐up growth and higher obesity risk in adulthood. Unlike the macrosomia seen in offspring of mothers with gestational diabetes, our model's reduced birth weight likely results from pancreatic β‐cell damage due to high glucose, causing maternal insulin deficiency [5]. This simulates an insulin deficiency similar to Type 1 diabetes, not insulin resistance. The deficiency leads to maternal malnutrition and restricted placental nutrient transport, resulting in fetal growth restriction [23]. Despite simulating insulin deficiency, this aligns with our study's focus on the impact of late‐pregnancy maternal hyperglycemia on offspring health. Behavioural, functional and morphological analysis revealed that GDM exposure during pregnancy led to reduced muscle mass, poor exercise performance, decreased muscle grip strength, muscle atrophy, abnormal muscle fibres, mitochondrial dysfunction and ectopic lipid deposition. Notably, exercise started after weaning improved muscle function and reversed metabolic dysfunction.

Transcriptomic analysis of GDM‐exposed offspring skeletal muscle at various stages (E18.5d, 3 weeks, 20 weeks) revealed significant changes in core transcriptional regulation. Common differential genes and pathways related to immune response, inflammation, chemotaxis and lipid metabolism were identified, suggesting that early GDM exposure reprograms immune‐inflammatory responses, leaving a lasting ‘imprint’ on postnatal development. GSEA analysis showed that GDM inhibits immune and inflammatory responses in fetal mice. However, in the skeletal muscle of adult GDM offspring, immune, inflammation and chemotaxis pathways were activated in weaned and adult skeletal muscle. Notably, exercise (GE group) down‐regulated inflammatory pathways, suggesting that exercise mitigates adverse skeletal muscle phenotypes in GDM offspring by reducing excessive inflammation.

The ce group showed upregulation of pathways directly related to skeletal muscle contraction and immune modulation, indicating exercise enhances muscle function and recovery through muscle activation and immune system regulation. In contrast, the GE group primarily enriched pathways related to glucose and lipid metabolism, suggesting that exercise improves skeletal muscle function in GDM offspring mainly by alleviating metabolic dysfunction. In this study, although we observed that exercise directly improved muscle mass, strength and metabolic function in GDM‐exposed offspring, we also recognize that exercise may exert its overall benefits by influencing the metabolic microenvironment of other tissues. Therefore, we suggest that future research should further explore how exercise promotes the improvement of skeletal muscle function through crosstalk between multiple organs and tissues, particularly via endocrine and metabolic signalling pathways. We further confirmed the analysis results obtained from the GSEA enrichment analysis via IPA analysis. Additionally, IPA analysis can present the dynamic change process of related signalling pathways and molecules more visually [24]. Increased immune cell infiltration was linked to inflammation‐related ectopic lipid deposition in skeletal muscle [25]. When ectopic lipids were deposited in skeletal muscle, pro‐inflammatory markers such as TNF‐α, IL‐1β and IFN‐γ increased, while anti‐inflammatory markers like IL‐10 were down‐regulated.

Peaks enrichment analysis of chromatin accessibility in GDM offspring skeletal muscle revealed reduced chromatin openness, while exercise increased this openness. Genes associated with differential chromatin accessibility in GDM offspring were mainly enriched in the Hippo signalling pathway, cAMP signalling pathway, TGF‐β signalling pathway and PI3K‐Akt signalling pathway. These signalling pathways played a crucial role in regulating the development of skeletal muscle, glucose and lipid metabolism functions as well as inflammation response [26, 27]. Additionally, aging‐related chronic metabolic diseases would impair skeletal muscle quality and fitness through the TGF‐β signalling pathway, while exercise training may enhance skeletal muscle mass and improve its function by downregulating inflammatory relevant molecules in this pathway.

Enrichment motif analysis across different developmental stages of GDM offspring skeletal muscle revealed a common TGATCA‐palindrome sequence in transcription factors. Interaction analysis highlighted the Junb‐Fos‐ATF3 complex, part of the AP‐1 family, as crucial in regulating abnormal skeletal muscle development. Expression analysis showed significantly high levels of AP‐1 family members, particularly Fos and Jun, in fetal‐stage GDM offspring skeletal muscle. AP‐1 family members were very sensitive to a variety of cellular stresses and quickly induced by even slight disturbance [28]. In response to the stress, AP‐1 family members display critical roles in the signalling transduction and transcriptional regulation to further regulate cell fate decision, therefore constituting one set of the most important and well characterized early response genes [29]. When skeletal muscle underwent different forms of atrophy, such as denervation, inactivity, starvation, diabetes, sepsis, acidosis and malignancy, the mRNA level of Junb was significantly up‐regulated [30]. In this study, we observed pathological phenotypes in the skeletal muscle of adult offspring of GDM, including abnormalities in muscle fibre and mitochondrial structures, as well as lipid deposition. These findings may be associated with the up‐regulation of Junb expression following a decrease in its chromatin accessibility. Conversely, previous studies have shown that a decrease in Junb content leads to rapid decreases in fibre diameter, resulting in skeletal muscle atrophy, while Junb overexpression can rescue this situation by stimulating overall protein synthesis [31]. This suggests that the elevated Junb levels in GDM contexts could represent a compensatory response or an alternative pathway affecting muscle homeostasis, which warrants further investigation. Although there has been no direct study on Fos and Junb in ectopic fat deposition in skeletal muscle under high‐glucose conditions, some studies suggest that Fos and Junb may play a role in regulating lipid metabolism. For example, Junb is known to be involved in adipocyte differentiation and lipid metabolism [32]. Long‐term aerobic exercise downregulates Fos in skeletal muscle via alleviating inflammation and enhancing satellite cell‐mediated muscle regeneration [33]. In our study, Fos and Junb exhibited significant changes in chromatin accessibility in the skeletal muscle of GDM‐exposed offspring and they may indirectly promote fat deposition by regulating the expression of genes related to lipid metabolism. Although our study does not directly demonstrate the role of Fos and Junb in ectopic fat accumulation in skeletal muscle, their potential roles in regulating inflammation and lipid metabolism merit further investigation. Ren et al. used ATAC‐seq and RNA‐seq technology to study the effects of high glucose exposure on human umbilical vein endothelial cells (HUVECs) [34]. They found that high glucose exposure leads to a decrease in chromatin accessibility and transcriptional level of Junb, whereas up‐regulating JunB can mitigate the effects of high glucose on vascular endothelial damage [34]. This is in contrast to the increased Junb expression observed in the skeletal muscle of GDM adult offspring in our study, suggesting potential tissue‐specific regulatory mechanisms in response to hyperglycemic conditions during development. Fos, as another major member of the AP‐1 family, played an important role in participating in cytokine secretion, immune regulation and the regulation of the inflammatory response [35, 36]. Similar to the expression trend of Junb, Fos expression levels were significantly higher in GDM offspring E18.5d skeletal muscle than in CTR offspring, while it was significantly lower than in CTR offspring in postnatal childhood and adulthood. Previous studies have shown that high glucose exposure increases the risk of atherosclerosis by up‐regulating c‐fos gene expression in smooth muscle cells of mouse vascular vessels activated by targeted SRE [37]. Fos expression in GDM offspring was significantly down‐regulated after leaving the intrauterine high glucose stress environment, which may be related to the excessive depletion of Fos in skeletal muscle in early life.

High‐glucose exposure in fetal mouse myoblasts triggers metabolic reprogramming, with upregulated metabolites like beta‐Alanine, EPA and Palmitic acid indicating adaptation to energy demands and inflammation, while downregulated metabolites like Lactate, Pantothenic acid and myo‐Inositol suggest disrupted glycolysis, oxidative metabolism and insulin signalling. This exposure also activates lipid‐related processes, confirmed by lipid accumulation in myoblasts via Oil Red O and Nile Red staining. These findings suggest high glucose promotes ectopic lipid deposition in skeletal muscle. Additionally, increased fos and Junb expression in myoblasts aligns with observations in mouse skeletal muscle. Further research is needed to understand how the AP‐1 family contributes to lipid deposition under high glucose and whether targeting these metabolites can alleviate muscle dysfunction.

## 5 Conclusion

In conclusion, our study showed that IUHG exposure led to adverse skeletal muscle phenotypes in adult offspring, including reduced muscle strength, lower muscle proportion, impaired exercise capacity, structural abnormalities and lipid deposition. ATAC‐Seq and RNA‐Seq analyses revealed significant changes in gene expression and chromatin accessibility, affecting processes like inflammation, immune response, chemotaxis and lipid deposition. The AP‐1 family transcription factors Fos and Junb may play a key role in these abnormalities. Exercise intervention showed potential in preventing muscle dysfunction, supporting the value of early screening and targeted interventions for GDM offspring.

## Funding

This work was supported by the National Key Research and Development Plan (2022YFC2703604, 2022YFC2703000, 2022YFC2703800, 2022YFC 2703500), National Natural Science Foundation of China (82271669, 82192864, 82171613, 82088102), Collaborative Innovation Program of Shanghai Municipal Health Commission (2020CXJQ01), Shanghai Clinical Research Center for Gynaecological Diseases (22MC1940200), Shanghai Frontiers Science Research Center of Reproduction and Development and the Fundamental Research Funds for the Central Universities 226‐2023‐00094.

## Ethics Statement

Animal procedures were all performed according to the ethical guidelines of the Animal Care and Use Committee of Zhejiang University (ZJU20210056).

## Conflicts of Interest

The authors declare no conflicts of interest.

## Supporting information


**Data S1:** Animal modelling protocol and offspring grouping.
**Data S2:** Offspring Exercise protocol.
**Data S3:** Skeletal muscle morphology detection methods.
**Data S4:** Detection of triglyceride (TG) content in skeletal muscle.
**Data S5:** Single‐cell RNA sequencing.
**Data S6:** RNA‐seq.
**Data S7:** ATAC‐seq.
**Data S8:** Quantitative real‐time polymerase chain reaction (qRT‐PCR).
**Data S9:** Western Blotting.
**Data S10:** Extraction of fetal mouse primary cells and in vitro high‐glucose intervention.
**Data S11:** Untargeted Metabolomic Analysis.
**Data S12:** Nile Red staining.

**Table S1:** Composition of the transposase mixture system.
**Table S2:** PCR enrichment reaction system.
**Table S3:** Primer sequencing for qPCR.
**Table S4:** Chemicals and reagents.
**Figure S1:** TG content in skeletal muscle of offspring at 20 weeks old. *n* = 6/group (every group from 3 litters), **p < 0.01, ***p < 0.005, One‐way ANOVA test was used and data were expressed as Mean ± SEM.
**Figure S2:** The quantification results of skeletal muscle morphology detection. (a) Muscle fibre cross‐sectional area (CSA) (μm^2^) was measured across different experimental groups. Statistical significance is indicated by asterisks, with *p < 0.05 and ***p < 0.001. (b) The percentage of Oil Red O + area, reflecting lipid accumulation, is shown for each group, with significant differences marked by ****p < 0.0001. (c) Impaired mitochondrial content (% of total mitochondria) was quantified, with statistical significance at **p < 0.01 and ****p < 0.0001 between the indicated groups. Data are presented as mean ± SEM, *n* = 4/group (every group from 4 litters).
**Figure S3:** GTT and ITT test results of offspring mice at adult period. (a) GTT test results of 8‐week‐old male offspring, *n* = 8/group (every group from 4 litters); (b) GTT test results of 12‐week‐old male offspring, *n* = 8/group (every group from 4 litters). CC vs. GC * *p* < 0.05, ** *p* < 0.01, GC vs. GE # *p* < 0.05, ## *p* < 0.01. (c) ITT of 16‐week‐old male offspring Experimental results, *n* = 8/group (every group from 4 litters); (d) ITT experimental results of 20‐week‐old offspring male mice, *n* = 8/group (every group from 4 litters), CC vs. GC *p<0.05, **p<0.01, ***p<0.005, GC vs. GE ##*p*<0.01, ###*p*<0.005. Two‐way ANOVA test was used and data were expressed as Mean ± SEM.
**Figure S4:** Microscopic examination of skeletal muscle single‐cell suspension and cell subpopulation proportions. (a) Microscopic examination (10×) of skeletal muscle single‐cell suspension from 20‐week‐old mice; (b) Single‐cell transcriptomic analysis of skeletal muscle at 20 weeks in control (CC) and gestational diabetes (GC) groups. The left panel shows UMAP plots of cell type clustering in skeletal muscle from the 20w CC and 20w GC. Different cell types are colour‐coded and labelled, including myonuclei of various types (Type I, Type IIa, Type IIb, Type IIx), FAPs, satellite cells, immune cells, smooth muscle cells, tenocytes, endothelial cells, neuromuscular junctions and myotendinous junctions. The right panel shows the relative percentages of each cell type in the control (CC) and gestational diabetes (GC) groups, with cell types colour‐coded according to their classification. The plots highlight the differences in cell type composition between the two groups. Each group contains one biological replicate, with each biological replicate consisting of cells pooled from three independent biological individuals, each derived from a different litter.
**Figure S5:** KEGG‐GO enrichment and GSEA analysis of the 20w ce vs. CC comparison. (a) KEGG‐GO enrichment analysis of differentially expressed genes in 20‐week‐old exercise (ce) vs. control (CC) offspring skeletal muscle. (b) Gene Set Enrichment Analysis (GSEA) of the regulation of striated muscle tissue development and adaptive immune response. Each biological replicate consists of 1 sample from a single mouse, with each mouse coming from a different litter, totally *n* = 3/group.
**Figure S6:** GSEA enrichment analysis of offspring skeletal muscle after intrauterine hyperglycemia exposure in transcriptional level. (a) GSEA Analysis of Skeletal Muscle in E18.5d Male Offspring with GDM group compared to CTR group. (b) GSEA Analysis of Skeletal Muscle in 3‐week‐old male offspring with GDM group compared to CTR group. (c) GSEA Analysis of Skeletal Muscle in 20‐week‐old male offspring with GDM group compared to CTR group. (d) GSEA Analysis of Skeletal Muscle in GDM exercise offspring compared with GDM group at 20 weeks old. Each biological replicate consists of 1 sample from a single mouse, with each mouse coming from a different litter, totally *n* = 3/group.
**Figure S7:** qPCR validation of differential expressed genes in skeletal muscle of 20‐week‐old offspring. (a) Immune response related differential genes qPCR validation; (b) Inflammatory response related differential genes qPCR validation; (c) Chemotaxis related differential genes qPCR validation. *N* = 5/group (each replicate derived from independent individuals from different litters), *p < 0.05, **p < 0.01, ***p < 0.005, ****p < 0.001.One‐way ANOVA test was used and data were expressed as Mean ± SEM.
**Figure S8:** Distribution of peaks across different elements of the genome. For E18.5d, the CTR‐F1 group consisted of 25 foetuses from 3 litters and the GDM‐F1 group consisted of 26 foetuses from 5 litters. Each biological replicate was composed of mixed samples from different litters (1:1 comparison). For the 3‐week‐old and 20‐week‐old groups, each biological replicate consisted of one mouse from a different litter, with each group having 2 biological replicates (2:2 comparison).
**Figure S9:** Lipid accumulation and AP‐1 protein expression in myoblasts exposed to high glucose (HG). (a) Nile Red and Oil Red O staining of myoblasts under control (CTRL) and high glucose (CTRL + HG) conditions；(b) Western blot analysis of Junb and Fos expression in myoblasts, (**p < 0.01, ***p < 0.005, ****p < 0.001). *N* = 4/group (every group from 4 litters, totally 8 litters, each litter of pups, from which myoblasts were extracted, constitutes one biological replicate). Quantitative data are shown as mean ± SEM. (c–f) The quantification bar charts of Nile Red, Oil Red O and WB, respectively.

## Data Availability

The paper and the supporting information present all data needed to evaluate the conclusions. Datasets used and/or analysed during the current study are available from the corresponding author on reasonable request.
