# Supplementary material for: Multi‐Omics Revealed the Effects of Intrauterine Hyperglycemia Exposure on the Development of Skeletal Muscle in Offspring
Source: J Cachexia Sarcopenia Muscle. 2026 Jan 22;17(1):e70177. doi: 10.1002/jcsm.70177 (PMC12827489; doi:10.1002/jcsm.70177)
Supplement: Supplementary file 1 — Data S1: Animal modelling protocol and offspring grouping. Data S2: Offspring Exercise protocol. Data S3: Skeletal muscle morphology detection methods. Data S4: Detection of triglyceride (TG) content in skeletal muscle. Data S5: Single‐cell RNA sequencing. Data S6: RNA‐seq. Data S7: ATAC‐seq. Data S8: Quantitative real‐time polymerase chain reaction (qRT‐PCR). Data S9: Western Blotting. Data S10: Extraction of fetal mouse primary cells and in vitro high‐glucose intervention. Data S11: Untargeted Metabolomic Analysis. Data S12: Nile Red staining. [file JCSM-17-e70177-s001.docx]

**Methods S1 Animal modeling protocol and offspring grouping**

Eight-week-old healthy female and male mice (Mus musculus, ICR) were purchased from Shanghai SLAC Laboratory Animal Co. Ltd(Shanghai, China, SCXK-2022-0004). Animals were housed in SPF conditions with a 12-hour light/dark cycle, ad libitum access to food/water, and 24°C temperature. After co-housing, vaginal plugs were examined to designate gestational day 0.5 (GD 0.5). Pregnant females were randomly assigned to the control (CTR) and intrauterine hyperglycemia (IUHG/GDM) groups. The GDM model was established using a two-stage β-cell disruption method.Immediately after birth, all offspring (both GDM and control groups) were cross-fostered to normoglycemic control dams to eliminate postpartum maternal diabetic effects such as altered lactation and nurturing behaviors. Cross-fostered offspring were subsequently weaned at three weeks of age. To ensure consistency in postnatal care, we cross-fostered all pups to healthy control dams immediately after birth and standardized the number of pups per foster mother to exactly 10 pups.After three weeks later of birth,mice were weaned and housed separately by gender,with 5 mice per cage, and were fed a normal diet with ad libitum access to water.We divided the F1 offspring into four groups based on whether exercise or not: CC (CTR-F1 non-exercise,control-control group),CE(CTR-F1 with exercise), GC(GDM-F1 non-exercise) and GE(GDM-F1 with exercise).

**Methods S2 Offspring Exercise protocol**

After weaning, mice in the CE and GE groups underwent a 5-day adaptation period on a treadmill, gradually increasing speed from 5 to 10 m/min and exercise duration from 20 to 50 minutes. Formal training began at 4 weeks, lasting 60 minutes per session, with warm-up, stable, and cool-down phases. Mice exercised 5 days per week for 16 weeks.

**Methods S3 Skeletal muscle morphology detection methods**

1. HE staining of skeletal muscle

Tibialis Anterior(TA) Muscle was fixed in formalin for 24 hours, then dehydrated, cleared in xylene, and embedded in paraffin. Sections (5μm) were cut, deparaffinized, and rehydrated. Hematoxylin stained nuclei, followed by eosin for the cytoplasm. Sections were dehydrated, cleared, and mounted with resin.

1. Oil Red O Staining of skeletal muscle

For Oil Red O staining, Tibialis Anterior(TA) Muscle was embedded in OCT, frozen, and sectioned at 8-10 microns. Sections were fixed in formalin, hydrated with isopropanol, and stained with Oil Red O for 10-15 minutes. After rinsing, sections were mounted with an aqueous medium and covered with coverslips.

1. TEM scaning of skeletal muscle

TA muscle tissue was fixed in 2.5% glutaraldehyde for 4 hours, stored at 4°C for 48 hours, and rinsed with PBS. Samples were fixed in osmium tetroxide, stained with uranyl acetate, and dehydrated in ethanol and acetone. After embedding, tissue was sectioned and stained for observation with a transmission electron microscope.

**Methods S4 Detection of triglyceride(TG) content in skeletal muscle**

To measure triglyceride content, 0.1g of tibialis anterior muscle was homogenized in a 1:1 mixture of isopropanol and heptane. The homogenate was then allowed to settle for 10 minutes. After settling, 100-200 μL of the supernatant was transferred to a new tube. The protein concentration was determined using a BCA assay and triglyceride content was normalized accordingly. The supernatant was heated, centrifuged, and the absorbance at 420 nm was measured after reacting with the appropriate working solution.

**Methods S5 Single-cell RNA sequencing**

Single-cell RNA sequencing library construction was performed using the 10x Genomics Chromium system: single cells were distributed into oil-phase microdroplets on a chip, followed by cell lysis, RNA reverse transcription, cDNA synthesis, and barcode labeling. After amplification, cDNA quality control was performed to confirm the library quality. Single-cell RNA sequencing was then conducted using the Illumina platform. Clustering analysis (such as t-SNE and UMAP) was used to identify different cell subpopulations, followed by feature gene analysis.The sequencing was performed by Lianchuan Biotechnology Co., Ltd.

**Methods S6 RNA-seq**

Total RNA was extracted from the skeletal muscle using the RNA fast200 kit (Feijie,Shanghai, China) according to the manufacturer's protocol. RNA quantity and purity were assessed using an ND-1000 Nanodrop (Thermo Fisher Scientific, Waltham, MA, USA). RNA purity was confirmed by A260/A280 and A260/A230 ratios of 1.8 and 2.0, respectively. The RNA integrity number (RIN) of each sample was measured using an Agilent 2100 Bioanalyzer (Agilent, Santa Clara, CA, USA). The RNA samples with a threshold RIN＞7.0 were subjected to further analyses. Nextgeneration RNA sequencing (RNA-seq) was carried out by Novogene Co., Ltd

**Methods S7 ATAC-seq**

The experiments involve transposition reaction, fragmentation product purification, PCR enrichment, PCR product purification, and ATAC library sequencing. For each sample, 5×10^5 cells are used. A transposase mix (Table S1) is added to the sample, followed by a 30-minute reaction at 37°C. The product is purified using the Vazyme TD 501 kit, and beads are added to the system for purification. The beads are washed with 80% ethanol and air-dried. After resuspending in distilled water, a PCR enrichment reaction is performed (Table S2). The PCR product is purified with VAHTS DNA Clean Beads and sequenced on an Illumina Hiseq PE150 sequencer, yielding 80-100 million paired-end reads. Quality control is done using FastQC, and clean reads are aligned to the mouse mm10 genome with Burrows-Wheeler Aligner.

**Methods S8 Quantitative real-time polymerase chain reaction (qRT-PCR)**

Quantitative real-time PCR (qRT-PCR) was used to verify mRNA expression levels of selected DEGs in offspring skeletal muscle at different developmental stages. Total RNA was reverse transcribed to cDNA using the PrimeScript™ RT reagent kit. PCR was performed with TB GREEN reagent, and primers are listed in **Table S3**. Expression levels were normalized to an internal control.

**Methods S9 Western Blotting**

Western blot was performed as previously described**[S1]**. Total protein of skeletal muscle and myoblast samples(30~50μ g) were loaded and separated using 10% polyacrylamide gels, and then the separated proteins were transferred onto the PVDF membrane. The membranes were incubated with antibodies which were listed in **Supplementary TableS4.** The band was visualized by chemiluminescence using an ECL kit (Thermo Fisher Scientific, Waltham, MA, USA, 34577).

**Methods S10 Extraction of fetal mouse primary cells and in vitro high-glucose intervention**

The extraction and isolation of fetal mouse myoblasts were performed based on modifications of our previously described methodology**[S2]**. Briefly, skeletal muscle was carefully excised, washed, and cut into small fragments. Collagenase II (Worthington Biochemical, USA, LS004176) was added to the tissue at a final concentration of 400U/ml in a 15ml tube for enzymatic digestion. The tube was incubated at 37°C with gentle agitation at maximum speed for 1 hour, with a 5-second vortex midway. After digestion, the solution was centrifuged at 1400×g for 5 minutes, and the supernatant was discarded. The pellet was resuspended in fresh media and gently pipetted several times using a sterile 10ml pipette. The resuspended material was then passed through pre-wet 70μm and 30μm strainers and centrifuged again at 1400×g for 5 minutes. The resulting pellet was resuspended in DMEM (American Type Culture Collection, USA, 30-2002) and plated onto a 6-cm dish. After 24 hours, the supernatant was collected and centrifuged at 930×g for 5 minutes. The pellet was resuspended in DMEM supplemented with bFGF (Thermo Fisher Scientific, USA, 100-18B) and plated onto dishes coated with 10% Matrigel (Corning, Inc., USA, 354234).After the cells adhere, treated with high-glucose (concentration: 35 mM) for 72 hours, and then cell samples will be collected for subsequent experiments.

**Methods S11 Untargeted Metabolomic Analysis**

Harvested myoblast cell samples were analyzed via LC-MS using a Vanquish UHPLC system coupled with an Orbitrap Q Exactive HF-X mass spectrometer. Differential metabolites were identified using OPLS-DA with thresholds of VIP ≥1 and P < 0.05. Data were processed using the Omicsmart platform(http://www.omicsmart.com).

**Methods S12 Nile Red staining**

Nile red staining experiments were performed using the lipid fluorescent staining kit(Solarbio,Cat:G1264), following the kit’s instructions. Cells were fixed with 4 % paraformaldehyde and then incubated with an appropriate volume of staining solution for 20 min away from light and re-stained using DAPI for 2 min. Images were collected by a Zeiss LSM 900 with Airyscan2microscope (Oberkochen, Germany) under a 485 nm excitation light source. Image-Pro Plus 6.0 software was used for data analysis.

**Supplementary Reference**

[S1] Mo, JY, Yan, YS, Lin, ZL, et al. Gestational diabetes mellitus suppresses fetal testis development in mice†. BIOL REPROD. 2022; 107 (1): 148-156.

[S2] Yan, YS, Mo, JY, Huang, YT, et al. Intrauterine hyperglycaemia during late gestation caused mitochondrial dysfunction in skeletal muscle of male offspring through CREB/PGC1A signaling. Nutr Diabetes. 2024; 14 (1): 56.
