# Supplementary material for: Multi‐Omics Revealed the Effects of Intrauterine Hyperglycemia Exposure on the Development of Skeletal Muscle in Offspring
Source: J Cachexia Sarcopenia Muscle. 2026 Jan 22;17(1):e70177. doi: 10.1002/jcsm.70177 (PMC12827489; doi:10.1002/jcsm.70177)
Supplement: Supplementary file 2 — Table S1: Composition of the transposase mixture system. Table S2: PCR enrichment reaction system. Table S3: Primer sequencing for qPCR. Table S4: Chemicals and reagents. Figure S1: TG content in skeletal muscle of offspring at 20 weeks old. n = 6/group (every group from 3 litters), **p < 0.01, ***p < 0.005, One‐way ANOVA test was used and data were expressed as Mean ± SEM. Figure S2: The quantification results of skeletal muscle morphology detection. (a) Muscle fibre cross‐sectional area (CSA) (μm2) was measured across different experimental groups. Statistical significance is indicated by asterisks, with *p < 0.05 and ***p < 0.001. (b) The percentage of Oil Red O + area, reflecting lipid accumulation, is shown for each group, with significant differences marked by ****p < 0.0001. (c) Impaired mitochondrial content (% of total mitochondria) was quantified, with statistical significance at **p < 0.01 and ****p < 0.0001 between the indicated groups. Data are presented as mean ± SEM, n = 4/group (every group from 4 litters). Figure S3: GTT and ITT test results of offspring mice at adult period. (a) GTT test results of 8‐week‐old male offspring, n = 8/group (every group from 4 litters); (b) GTT test results of 12‐week‐old male offspring, n = 8/group (every group from 4 litters). CC vs. GC * p < 0.05, ** p < 0.01, GC vs. GE # p < 0.05, ## p < 0.01. (c) ITT of 16‐week‐old male offspring Experimental results, n = 8/group (every group from 4 litters); (d) ITT experimental results of 20‐week‐old offspring male mice, n = 8/group (every group from 4 litters), CC vs. GC *p<0.05, **p<0.01, ***p<0.005, GC vs. GE ##p<0.01, ###p<0.005. Two‐way ANOVA test was used and data were expressed as Mean ± SEM. Figure S4: Microscopic examination of skeletal muscle single‐cell suspension and cell subpopulation proportions. (a) Microscopic examination (10×) of skeletal muscle single‐cell suspension from 20‐week‐old mice; (b) Single‐cell transcriptomic analysis of skeletal muscle at 20 weeks i [file JCSM-17-e70177-s002.docx]

**Supplymentary materials**

**Table S1 Composition of the transposase mixture system**

| Reaction component | Dosage |
| --- | --- |
| 5×TTBL buffer | 10 μl |
| TTE mix | 5 μl |
| 1% Digitonin | 0.5 μl |
| 10% Tween-20 | 0.5 μl |
| ddH2O | 34 μl |

**Table S2 PCR enrichment reaction system**

| Reaction component | Dosage |
| --- | --- |
| Purified fragmented DNA | 24 μl |
| 5×TAB | 10 μl |
| PPM | 5 μl |
| P5 Primer X | 5 μl |
| P7 Primer X | 5 μl |
| TAE | 1μl |

**Table S3 Primer sequencing for qPCR**

| Gene | Sequencing(5’-3’) |
| --- | --- |
| *18S* | F:GGCTGTATTCCCCTCCATCG R:CCAGTTGGTAACAATGCCATGT |
| *Il1a* | F: TCTGTCCCGGATCTACCTTG R: GTAGAATCCAAGCGCGAAAC |
| *Il1b* | F: GGATGACAGGCTTGCAGCTAT R: TTTGTGCAACTAGGAACGTAAGTCG |
| *Cxcr6* | F: GCTGAGAGCTTGAAGCACAAGA R: TTGAGATGCCCAGAGGATCAC |
| *Tlr1* | F：CCTAGCAGTTATCACAAGCTCAAA R：TCTTTTCCTTGGGCCATTC |
| *Tlr2* | F：CGTTCTCTCAGGTGACTGCTC R：TCTCCTTTGGATCCTGCTTG |
| *Tlr8* | F：AGCACTTCCCTCAGGAAGATT R：AGCACCTTCAGATGAGGCATA |
| *Tlr13* | F：TGCTCGGAAACCTACCCAAG R：GAAGGAGAGCTGGCACTTGT |
| *Cd80* | F：GGGCGTACACTTTCCCTTCTC R：GCCTGACCTACTGCTTTGCC |
| *Cd28* | F：GAAGATGCAAGGTGGTGAGATTG R：CCTGGCCCAAAACATGCT |
| *Cd86* | F：AGTGATCGCCAACTTCAGTGAACC R：GGTGACCTTGCTTAGACGTGCAG |
| *Itgb7* | F：CAGCTACATGCACGGTCTGT R：ACACAATGTCGGGTTTCACA |
| *Il10* | F：GCTCTTACTGACTGGCATGAG R：CGCAGCTCTAGGAGCATGTG |
| *Ly86* | F：ACAGTGCCACACGAGGTACA R：ACAGTGCCACACGAGGTACA |
| *Nlrp3* | F：AGGAGAATGGACCTGCAAGC R：TCTACCATCATCCAGCCTTGG |
| *Cxcl3* | F：TGAATCCGGAATCTAAGACCATCAA R：AGGACTAGCCATCCACTGGGTAAAG |
| *Cxcl5* | F：CTGCGGCAGCGTGAACAGCAAC R：ATCTTGTCCACAATGAGCCTCC |
| *Ccl3* | F：CTCTGCAACCAGGTCCTCTC R：TTTCTGGACCCACTCCTCAC |
| *Ccl4* | F：CCAATGGGCTCTGACCCTCCC R：CTTCTGTGCTCCAGGGTTCTC |
| *Ccl6* | F：TTGCTCAGCCAGTTGGGATT R：AAAGTCCTGGACCCACTTCTGT |
| *Ccl9* | F：CCTCACCAAGCTGGAGAGGCCCT R：CTGAGGCTCACGTCACCAAGTC |
| *Ccl12* | F：ACCATCAGTCCTCAGGTATTGG R：TTCCGGACGTGAATCTTCTG |
| *Ccl20* | F：GCCTCTCGTACATACAGACGC R：CCAGTTCTGCTTTGGATCAGC |

**Table S4 Chemicals and reagents**

| **Chemicals and reagents** | **Manufacturer/Supplier** |
| --- | --- |
| Insulin | Procell(Cat .No.PB180432),Wuhan China |
| D-glucose | Sigma-Aldrich(Cat .No.G5767),USA |
| STZ | Sigma-Aldrich(Cat .No.18883-66-4),USA |
| Citrate Buffer | Solarbio(Cat .No.C1013),Beijing China |
| FOS-antibody | SYSY company(Cat .No.226-017),Germany |
| Junb-antibody | Proteintech (10486-1-AP),USA |
| Nile red kits | Solarbio(Cat .No.G1264),Beijing China |
| Oil O red kits | Solarbio(Cat .No.G1261),Beijing China |

**Figure S1**

**Figure S2**


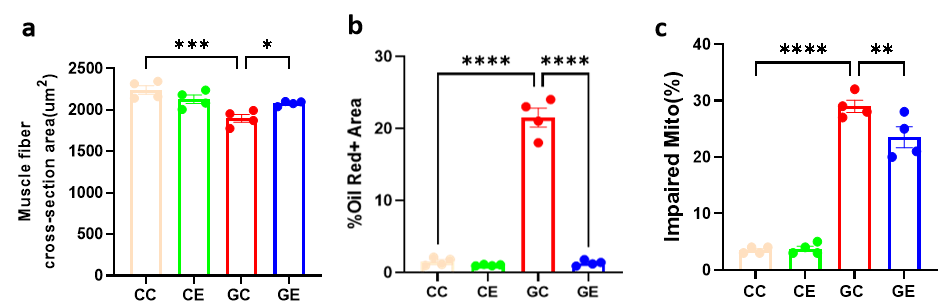


**Figure S3**


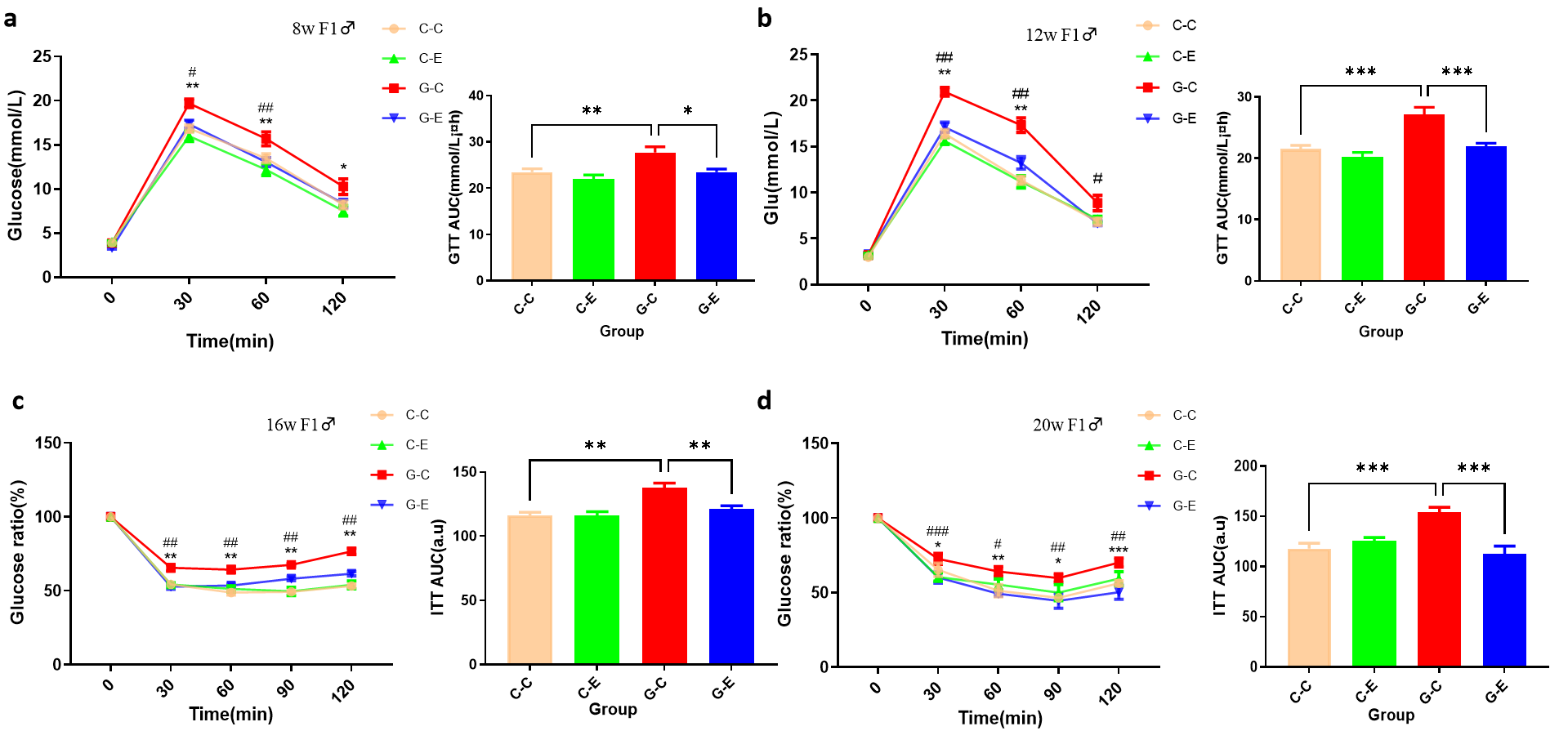


**Figure S4**


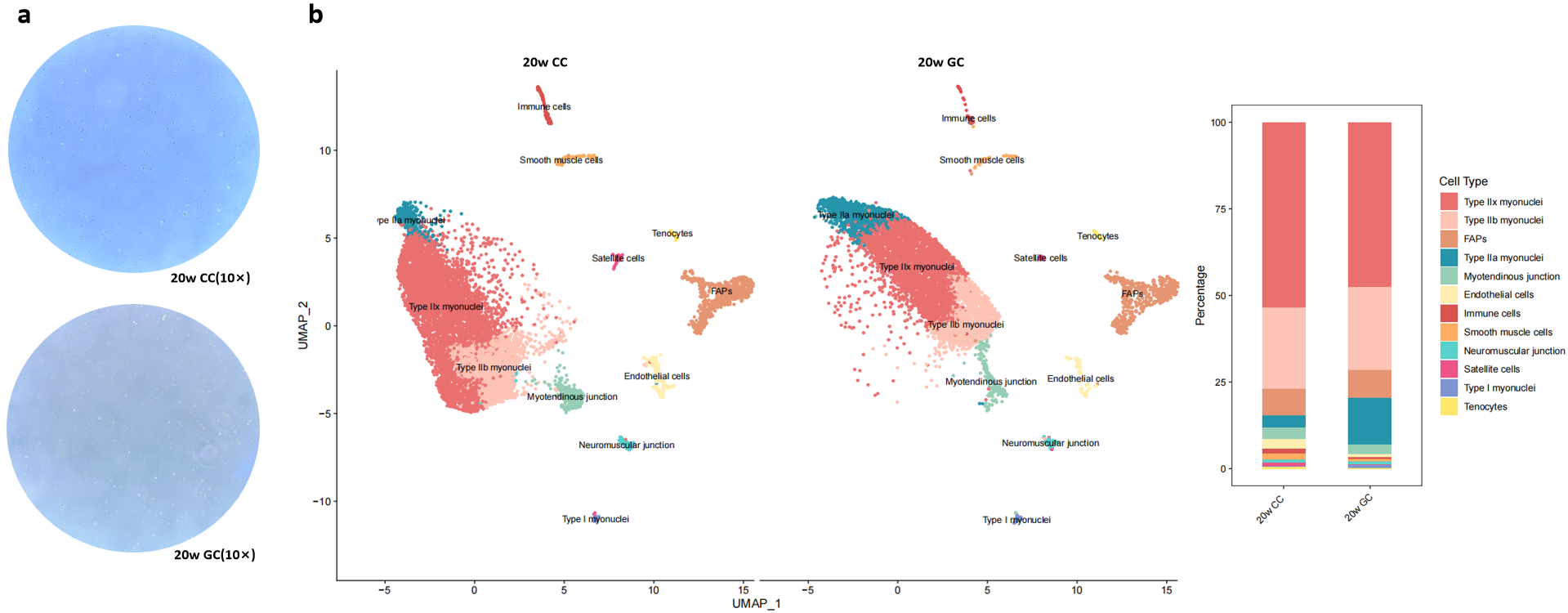


**Figure S5**


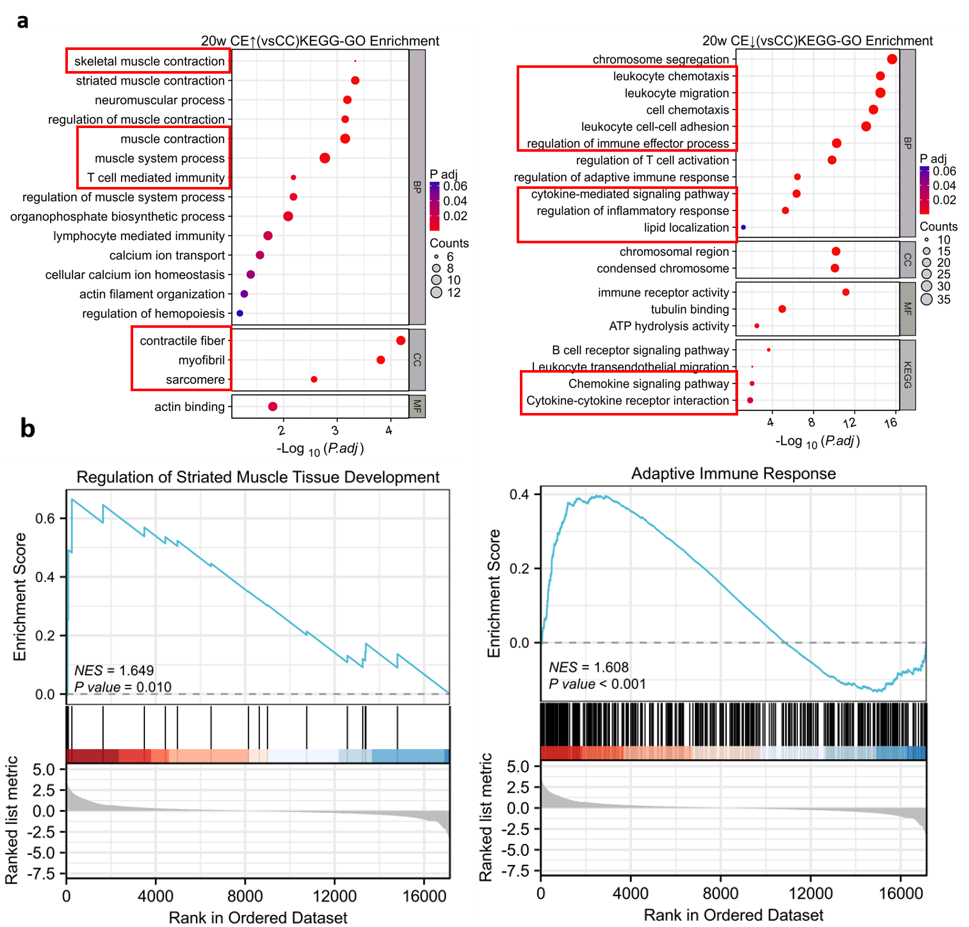


**Figure S6**


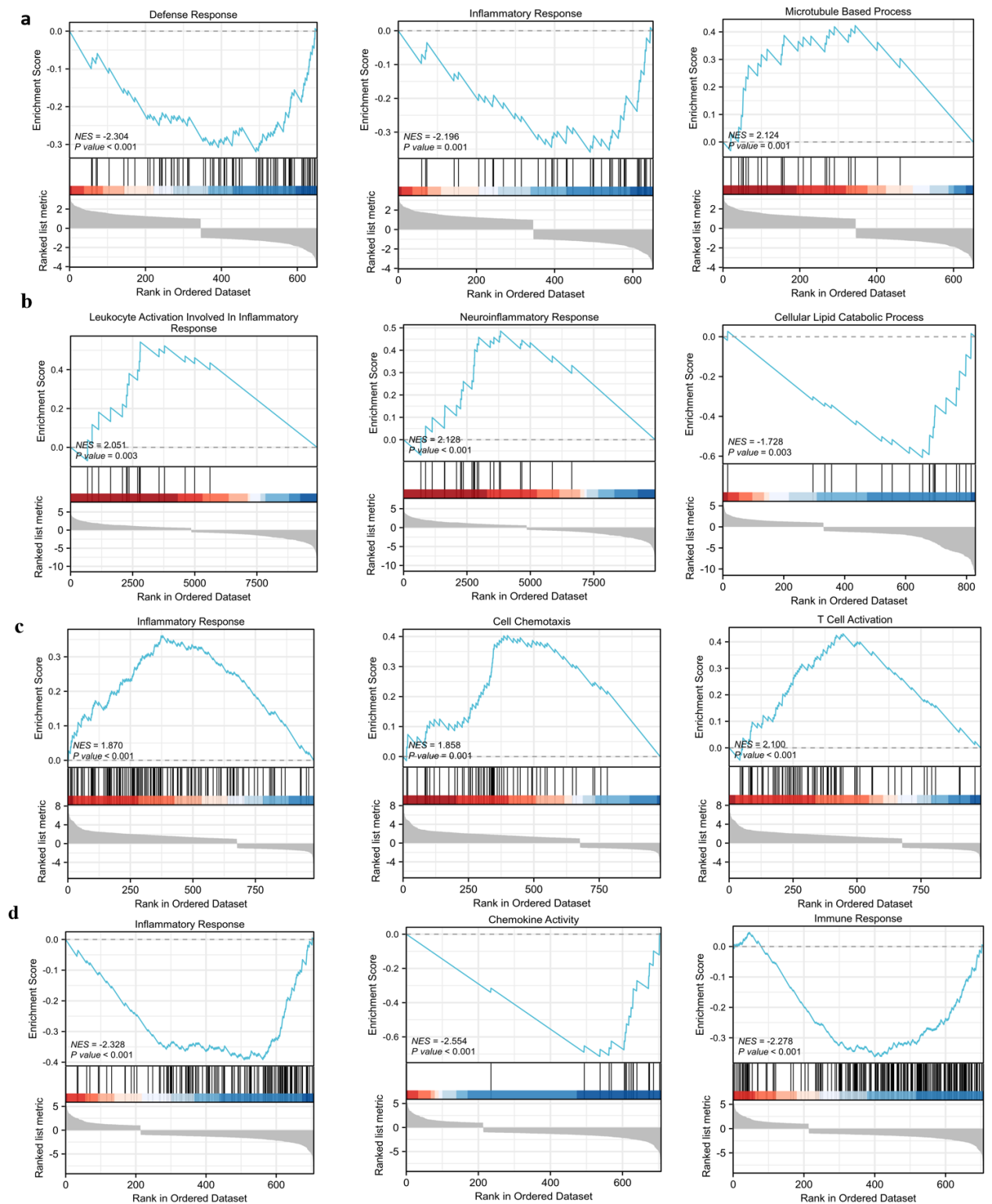


**Figure S7**


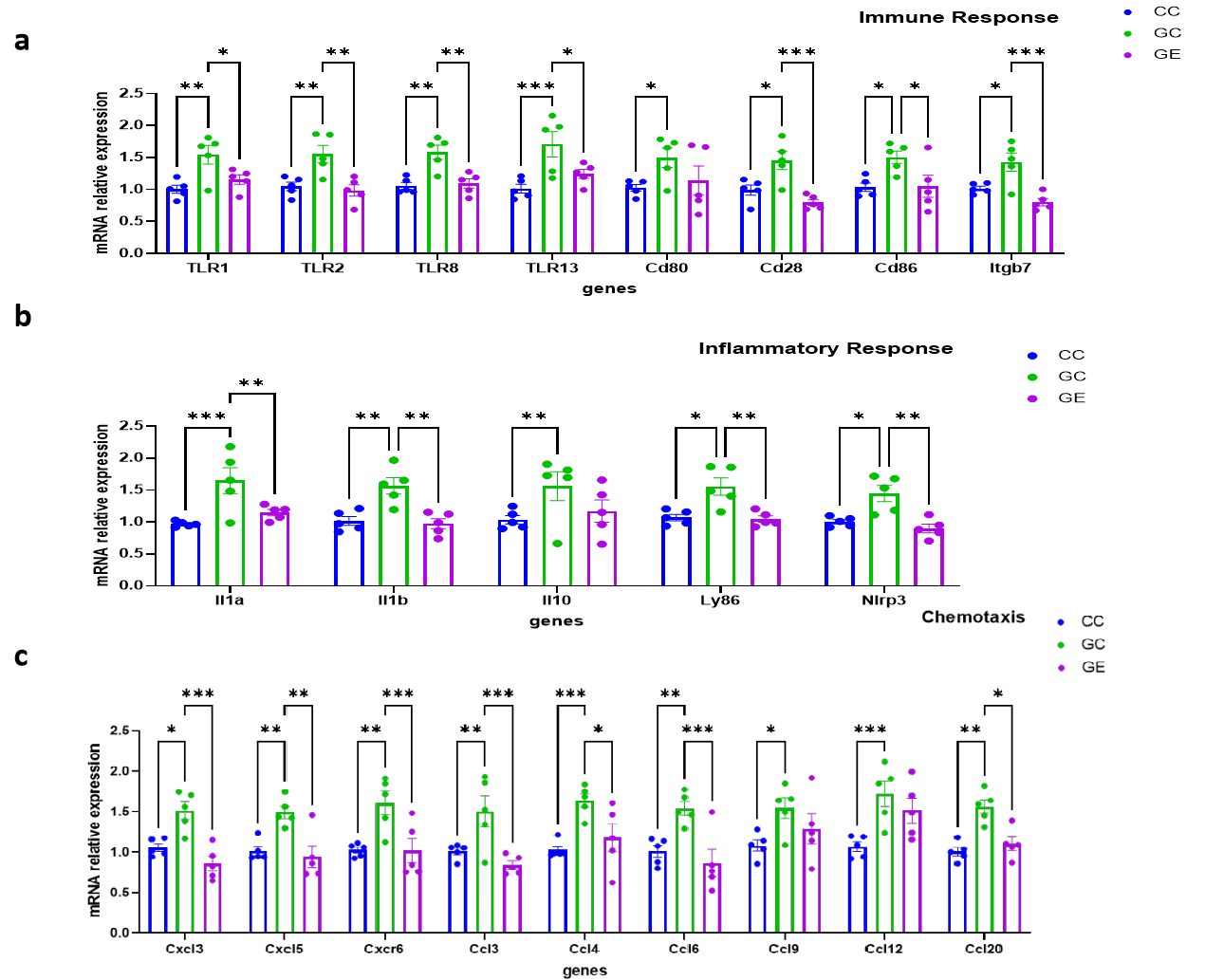


**Figure S8**


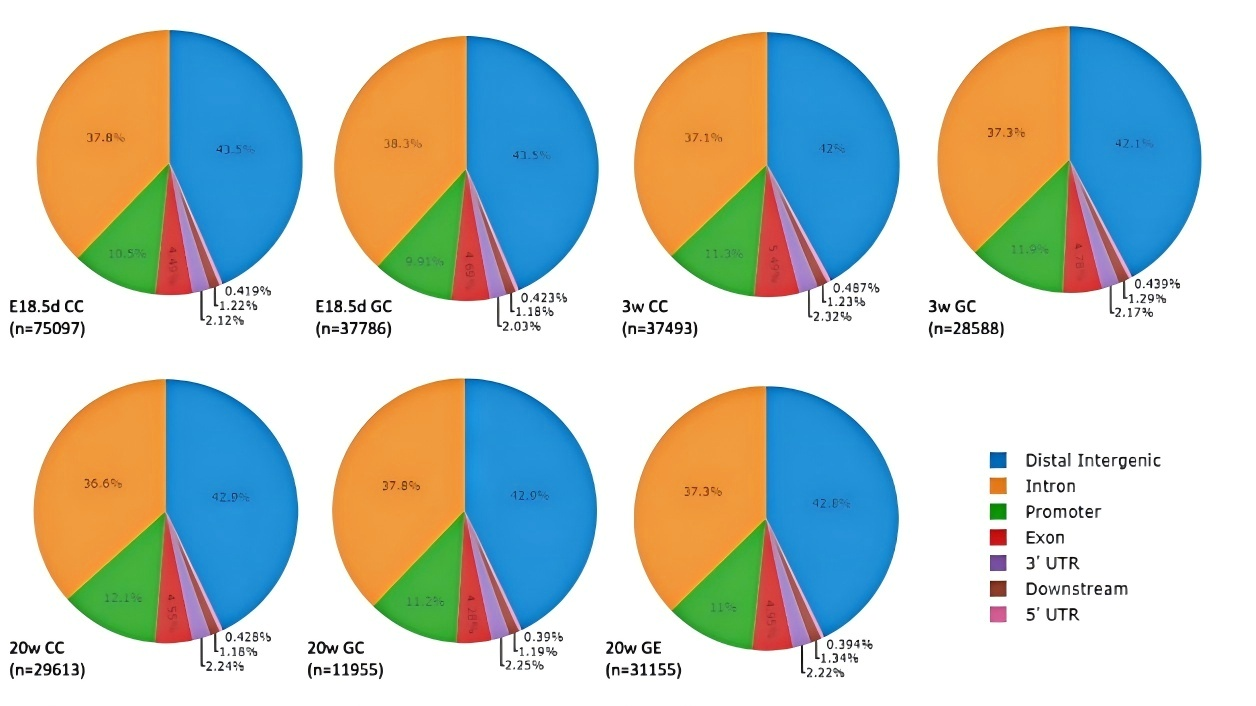


**Figure S9**


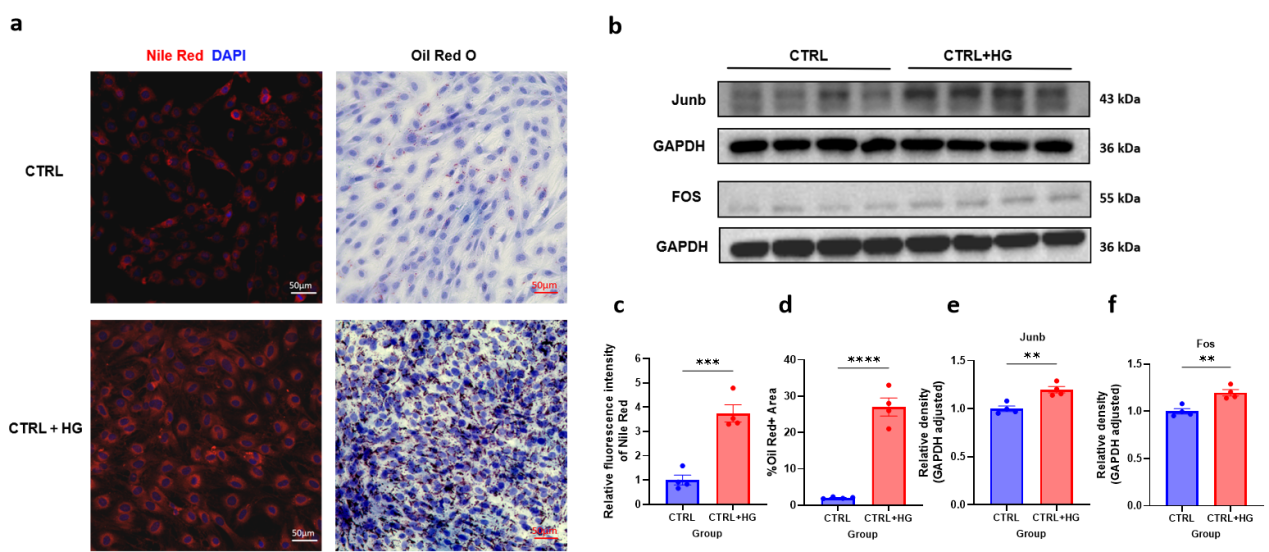


**Supplementary Figure legends:**

**Fig S1 TG content in skeletal muscle of offspring at 20-week-old**. n=6/group**(every group from 3 litters)**,***p*＜0.01, ****p*＜0.005,One-way ANOVA test was used, and data were expressed as Mean±SEM.

**Fig S2 The quantification results of skeletal muscle morphology detection.**(a) Muscle fiber cross-sectional area(CSA) (µm²) was measured across different experimental groups. Statistical significance is indicated by asterisks, with **p* < 0.05 and ****p* < 0.001. (b) The percentage of Oil Red O+ area, reflecting lipid accumulation, is shown for each group, with significant differences marked by *****p* < 0.0001. (c) Impaired mitochondrial content (% of total mitochondria) was quantified, with statistical significance at ***p* < 0.01 and *****p* < 0.0001 between the indicated groups. Data are presented as mean ± SEM, **n=4/group(every group from 4 litters)**.

**Fig S3 GTT and ITT test results of offspring mice at adult period.**(a) GTT test results of 8-week-old male offspring, n=8/group**(every group from 4 litters)**; (b) GTT test results of 12-week-old male offspring, n=8/group**(every group from 4 litters)**. CC vs GC * p< 0.05, ** p< 0.01, GC vs GE # p < 0.05, ## p< 0.01. (c) ITT of 16-week-old male offspring Experimental results, n=8/group**(every group from 4 litters)**; (d) ITT experimental results of 20-week-old offspring male mice, n=8/group**(every group from 4 litters)**,CC vs GC *p＜0.05, **p＜0.01, ***p＜0.005, GC vs GE ##p＜0.01, ###p＜0.005. Two-way ANOVA test was used, and data were expressed as Mean±SEM.

**Fig S4 Microscopic examination of skeletal muscle single-cell suspension and cell subpopulation proportions.** (a) Microscopic examination (10×) of skeletal muscle single-cell suspension from 20-week-old mice; (b) Single-cell transcriptomic analysis of skeletal muscle at 20 weeks in control (CC) and gestational diabetes (GC) groups. The left panel shows UMAP plots of cell type clustering in skeletal muscle from the 20w CC and 20w GC. Different cell types are color-coded and labeled, including myonuclei of various types (Type I, Type IIa, Type IIb, Type IIx), FAPs, satellite cells, immune cells, smooth muscle cells, tenocytes, endothelial cells, neuromuscular junctions, and myotendinous junctions. The right panel shows the relative percentages of each cell type in the control (CC) and gestational diabetes (GC) groups, with cell types color-coded according to their classification. The plots highlight the differences in cell type composition between the two groups.**Each group contains one biological replicate, with each biological replicate consisting of cells pooled from three independent biological individuals, each derived from a different litter.**

**Fig S5: KEGG-GO enrichment and GSEA analysis of the 20w CE vs CC comparison.**(a) KEGG-GO enrichment analysis of differentially expressed genes in 20-week-old exercise (CE) vs control (CC) offspring skeletal muscle. (b) Gene Set Enrichment Analysis (GSEA) of the regulation of striated muscle tissue development and adaptive immune response. **Each biological replicate consists of 1 sample from a single mouse, with each mouse coming from a different litter,totally n=3/group.**

**Fig S6 GSEA enrichment analysis of offspring skeletal muscle after intrauterine hyperglycemia exposure in transcriptional level**. (a) GSEA Analysis of Skeletal Muscle in E18.5d Male Offspring with GDM group compared to CTR group.(b) GSEA Analysis of Skeletal Muscle in 3-week-old male offspring with GDM group compared to CTR group.(c) GSEA Analysis of Skeletal Muscle in 20-week-old male offspring with GDM group compared to CTR group.(d) GSEA Analysis of Skeletal Muscle in GDM exercise offspring compared with GDM group at 20-week-old. **Each biological replicate consists of 1 sample from a single mouse, with each mouse coming from a different litter,totally n=3/group.**

**Fig S7 qPCR validation of differential expressed genes in skeletal muscle of 20-week-old offspring.**(a)Immune response related differential genes qPCR validation;(b)Inflammatory response related differential genes qPCR validation; (c)Chemotaxis related differential genes qPCR validation.N=5/group **(each replicate derived from independent individuals from different litters )**,*p＜0.05, **p＜0.01, ***p＜0.005, ****p＜0.001.One-way ANOVA test was used, and data were expressed as Mean±SEM.

**Fig S8 Distribution of peaks across different elements of the genome.For E18.5d, the CTR-F1 group consisted of 25 fetuses from 3 litters, and the GDM-F1 group consisted of 26 fetuses from 5 litters. Each biological replicate was composed of mixed samples from different litters (1:1 comparison). For the 3-week-old and 20-week-old groups, each biological replicate consisted of one mouse from a different litter, with each group having 2 biological replicates (2:2 comparison).**

**Fig S9 Lipid accumulation and AP-1 protein expression in myoblasts exposed to high glucose (HG).**(a) Nile Red and Oil Red O staining of myoblasts under control (CTRL) and high glucose (CTRL + HG) conditions；(b) Western blot analysis of Junb and Fos expression in myoblasts, **p < 0.01, ***p < 0.005,****p < 0.001) . **N=4/group (every group from 4 litters, totally 8 litters,each litter of pups, from which myoblasts were extracted, constitutes one biological replicate).**Quantitative data are shown as mean ± SEM. (c-f) The quantification bar charts of Nile Red, Oil Red O, and WB, respectively.
